# Beyond the Apnea-Hypopnea Index: Cardiovascular and Metabolic Phenotyping of Hypertensive Patients with Obstructive Sleep Apnea—A Narrative Review

**DOI:** 10.3390/jcm15197461

**Published:** 2026-09-25

**Authors:** Georgiana-Mihaela Cebuc Simeanu, Adina Dorina Glodeanu, Diana-Cristina Protasiewicz-Timofticiuc, Alina-Liliana Popa, Adina Mitrea, Ion-Cristian Efrem, Diana Clenciu, Adina-Andreea Mirea, Denisa-Maria Mitroi, Ioana-Gabriela Dragne, Bianca-Florentina Pîrvu, Delia-Viola Reurean-Pintilei, Beatrice Elena Vladu, Ana-Maria Efrem, Ștefania-Liana Nicolescu, Mircea-Catalin Fortofoiu

**Affiliations:** 1 Doctoral School, University of Medicine and Pharmacy, 200349 Craiova, Romania; georgiana.simeanu@umfcv.ro (G.-M.C.S.); denisa_maria2@yahoo.com (D.-M.M.); gabriela.dragne@umfcv.ro (I.-G.D.); bianca.pirvu@umfcv.ro (B.-F.P.); catalin.fortofoiu@umfcv.ro (M.-C.F.); 2 Department of Internal Medicine, Faculty of Dentistry, University of Medicine and Pharmacy of Craiova, 200349 Craiova, Romania; glodeanuadina@yahoo.com (A.D.G.); cristian.efrem@umfcv.ro (I.-C.E.); 3 Department of Diabetes, Nutrition and Metabolic Diseases, Faculty of Midwives and Nursing, University of Medicine and Pharmacy of Craiova, 200349 Craiova, Romania; 4 Department of Diabetes, Nutrition and Metabolic Diseases, Faculty of Medicine, University of Medicine and Pharmacy of Craiova, 200349 Craiova, Romania; adina.mitrea@umfcv.ro (A.M.); diana.clenciu@umfcv.ro (D.C.); 5 Department of Oro-Dental Prevention and Oral Health, Faculty of Dentistry, University of Medicine and Pharmacy of Craiova, 200349 Craiova, Romania; adina.mirea@umfcv.ro; 6 Research Centre in Dental Medicine, University of Medicine and Pharmacy of Craiova, 200349 Craiova, Romania; 7 Department of Medical-Surgical and Complementary Sciences, Faculty of Medicine and Biological Sciences, “Ștefan cel Mare” University, 720229 Suceava, Romania; delia.pintilei@usm.ro; 8 Faculty of Medicine, University of Medicine and Pharmacy of Craiova, 200349 Craiova, Romania; beatricevladu75@gmail.com; 9 Faculty of Medicine, University of Medicine and Pharmacy “Iuliu Haţieganu” Cluj-Napoca, 400012 Cluj-Napoca, Romania; anamaref23@gmail.com; 10 Department of Diabetes, Nutrition and Metabolic Diseases, Clinical Municipal Hospital Phylanthropy, 200143 Craiova, Romania; lia.nicolescu@yahoo.com; 11 Department of Medical Semiology, Faculty of Medicine, University of Medicine and Pharmacy of Craiova, 200349 Craiova, Romania

**Keywords:** obstructive sleep apnea, hypertension, cardiovascular phenotyping, hypoxic burden, ambulatory blood pressure monitoring, target-organ damage, insulin resistance, MASLD, precision medicine

## Abstract

Obstructive sleep apnea (OSA) is strongly associated with hypertension, yet the apnea–hypopnea index (AHI), while it defines the presence and frequency of respiratory events, predicts cardiovascular risk less reliably in the individual patient than metrics that incorporate the depth and duration of hypoxic exposure. Patients with comparable AHI values can differ substantially in nocturnal hypoxemia, sympathetic activation, ambulatory blood pressure behavior, and target-organ damage, exposing a persistent gap between respiratory classification and cardiovascular prognosis. This review synthesizes current evidence on the mechanisms linking OSA to hypertension, intermittent hypoxia, chemoreflex-mediated sympathetic overactivity, renin–angiotensin–aldosterone system activation, hypoxia-associated insulin resistance and examines emerging metrics, including hypoxic burden, oxygen desaturation index, and ambulatory blood pressure monitoring, that capture cardiovascular risk more accurately than AHI alone. We describe six cardiovascular phenotypes of hypertension in OSA: resistant, nocturnal, non-dipping/reverse-dipping, masked, and morning-surge hypertension, alongside hypertension-mediated target-organ damage, each with distinct mechanisms, prognosis, and treatment response. Because intermittent hypoxia and sympathetic activation are also implicated in insulin resistance and MASLD, this shared metabolic pathway is integrated throughout rather than treated as an incidental comorbidity. Building on this evidence, we propose a phenotype-oriented clinical framework spanning cardiovascular screening, multidomain phenotyping, risk stratification, and personalized therapy intended to complement, not replace, AHI-based diagnosis and current hypertension guidelines. This framework is presented as a conceptual, expert-derived proposal intended to structure clinical reasoning, and it has not been derived from or validated in an independent cohort. We conclude with future directions, including digital biomarkers, multimodal imaging, and artificial intelligence-assisted phenotyping, as pathways toward precision cardiovascular medicine in hypertensive patients with OSA.

## 1. Introduction

Obstructive sleep apnea (OSA), systemic hypertension, and metabolic dysfunction-associated steatotic liver disease (MASLD) form a distinct cardiometabolic triad. Rather than acting as separate entities, these conditions develop against a shared background of metabolic syndrome and insulin resistance [1,2,3]. Visceral obesity, glucose intolerance, and overt type 2 diabetes mellitus frequently accompany all three disorders [3,4]. Repeated upper airway collapse during sleep contributes to intermittent hypoxia and sleep fragmentation. These nocturnal events trigger sympathetic surges, oxidative stress, and renin-angiotensin-aldosterone system activation, which elevate blood pressure and accelerate hepatic fat accumulation [5,6,7]. In parallel, hepatic steatosis and insulin resistance impair systemic vascular compliance, creating a self-reinforcing loop that worsens long-term cardiovascular outcomes [7,8].

The epidemiological overlap across these disorders is striking. Between one-third and one-half of hypertensive individuals have concomitant OSA, a rate that exceeds 70% among those with resistant hypertension [1,2]. At the same time, MASLD affects more than 30% of adults worldwide, reaching over 70% in people with metabolic syndrome, type 2 diabetes, or severe sleep apnea [3,7,8]. Untreated OSA impairs blood pressure control despite multi-drug therapy and accelerates liver injury from simple steatosis to steatohepatitis and fibrosis [4,7,8]. Conversely, persistent hypertension, poor glycemic control, and advanced liver fibrosis amplify vascular damage and increase the risk of major adverse cardiovascular events. Current clinical guidelines suggest screening for OSA in selected hypertensive populations. However, diagnostic protocols rarely incorporate glycemic or hepatic metabolic screening into routine sleep workups.

Despite progress in understanding these mechanisms, daily practice still relies almost exclusively on the apnea-hypopnea index (AHI) to grade disease severity. The AHI counts respiratory events per hour of sleep. This makes it simple to calculate and highly reproducible, but it also means that the index does not, by design, reflect the physiological consequences of those events. Two patients with an identical AHI of 30 events per hour often show vastly different degrees of nocturnal desaturation, insulin resistance, autonomic instability, target-organ damage, and long-term cardiovascular risk [4,6]. A single event count cannot reflect the biological stress imposed on the vascular wall, pancreatic beta cells, or liver tissue.

This clear limitation shifted research toward parameters that better quantify biological burden. Among these, hypoxic burden has received the most extensive investigation, because it accounts for both the depth and the duration of nocturnal desaturations rather than for event frequency alone. Other metrics, including the oxygen desaturation index (ODI), sleep time below 90% oxygen saturation (T90), heart rate variability, continuous glucose monitoring indices, and 24-h ambulatory blood pressure monitoring (ABPM), have shown closer associations with cardiovascular injury and glycemic variability than the AHI in several cohorts [4,5,6,7]. The magnitude of this advantage varies by outcome, however, and none of these metrics has yet been standardized for routine clinical use, points developed in Section 3.2 and returned to in the limitations.

Similarly, hypertension in patients with sleep apnea is far from uniform. Non-dipping and reverse-dipping nocturnal blood pressure profiles, isolated nocturnal hypertension, masked hypertension, and exaggerated morning blood pressure surges represent distinct vascular phenotypes. Each subtype carries unique prognostic implications for organ damage, especially when combined with type 2 diabetes or severe insulin resistance [1,2,8]. Yet diagnostic pathways keep these conditions in separate silos. This separation helps explain why therapeutic responses to continuous positive airway pressure (CPAP) vary widely among patients who appear similar on paper.

Precision medicine demands a broader perspective. Rather than viewing OSA purely as a mechanical respiratory problem, we need an integrated, phenotype-driven strategy. Combining respiratory severity with nocturnal hypoxic burden, ABPM patterns, autonomic responses, glycemic status, and MASLD risk markers provides a fuller picture of the patient’s cardiovascular phenotype.

The clinical challenge has evolved from diagnosing OSA to identifying hypertensive patients at the highest cardiovascular risk. Integrating respiratory indices with cardiovascular phenotyping may improve risk assessment and patient characterization; whether phenotype-guided treatment improves clinical outcomes remains to be determined, and this qualification applies throughout the review.

We evaluate the mechanistic links connecting hypertension, OSA, and MASLD within the spectrum of insulin resistance and metabolic syndrome. We discuss why traditional AHI-based staging falls short in predicting cardiovascular and metabolic risk. Finally, we propose a cardiovascular-metabolic phenotyping framework that integrates sleep metrics, blood pressure profiles, metabolic indices, and hepatic risk markers. This framework is conceptual and expert-derived: it has not been developed from patient-level data, nor calibrated or validated in an independent cohort, and it is offered as a way of structuring clinical reasoning rather than as a validated instrument. The conceptual trajectory of this review, from the physiological limitations of the AHI, through the mechanisms linking intermittent hypoxia to cardiovascular injury, to the resulting clinical phenotypes and their personalized management, is summarized graphically in Figure 1 and developed in detail in the sections that follow.

## 2. Approach to the Literature

This article is a narrative review. Its purpose is to synthesize and interpret a heterogeneous body of evidence spanning sleep medicine, hypertension, cardiovascular imaging and metabolic disease, and to propose a conceptual framework on that basis. It was not designed or conducted as a systematic review, and the account that follows describes the approach taken rather than a predefined protocol.

The literature was identified through searches of PubMed/MEDLINE, Embase, Scopus and the Cochrane Library covering publications up to June 2026, using combinations of Medical Subject Headings and free-text terms corresponding to the principal domains developed in this review, namely obstructive sleep apnea, hypertension, cardiovascular risk and phenotyping, hypoxic burden and related oximetry indices, ambulatory blood pressure monitoring and its derived phenotypes, hypertension-mediated target-organ damage, insulin resistance, MASLD, and CPAP. Searching was iterative rather than executed as a single fixed query: as the argument of the review developed, additional searches were performed on specific questions, and further sources were identified through the reference lists of retrieved articles and through current hypertension and sleep-medicine guideline documents.

Sources were selected by the authors on the basis of relevance to the domains above and of methodological standing, with priority given to large prospective cohorts, randomized controlled trials, systematic reviews and meta-analyses, scientific statements and current guidelines, supplemented by mechanistic and experimental work where the pathophysiological argument required it. No predefined eligibility criteria were applied, screening was not performed in duplicate, and no formal risk-of-bias appraisal was undertaken. Only publications in English were considered. Where the evidence on a given point is limited, inconsistent or derived from a single study, this is stated at that point in the text rather than handled through a quality-scoring instrument.

The implications of this approach for the interpretation of the review are considered in the limitations.

## 3. Why the Apnea–Hypopnea Index Is No Longer Sufficient

### 3.1. The Limitations of Respiratory Event Counting

For over four decades, clinicians have relied on the apnea-hypopnea index (AHI) as the primary diagnostic metric for obstructive sleep apnea (OSA). The AHI provides a single numerical value: the average number of respiratory disruptions occurring per hour of sleep. It dictates diagnostic cutoffs, guides therapeutic decisions, and serves as the main entry criterion in major clinical trials [9]. Yet counting airway collapses tells us very little about what happens inside the body during those collapses. Relying on event frequency alone therefore leaves the downstream biological impact unmeasured. Consequently, although the AHI is associated with cardiovascular and metabolic outcomes at the population level, it predicts them less reliably in the individual patient than metrics that incorporate the depth and duration of hypoxic exposure.

The core limitation lies in how the index is calculated. Every apnea or hypopnea counts equally toward the total score. A 10-s partial airway obstruction accompanied by a minor oxygen dip receives the exact same weight as a 60-s complete occlusion that causes profound hypoxemia, intense sympathetic discharge, and a sharp spike in blood pressure. The AHI does not incorporate event duration, the depth of desaturation, the sleep stage in which events occur, body position, or individual autonomic reactivity [5,10,11]. This limitation is particularly consequential in hypertensive patients with metabolic syndrome or MASLD [12,13]. In these individuals, systemic damage depends on cumulative physiological stress rather than event frequency [14]. Consider two patients who both present with an AHI of 30 events per hour. The first patient experiences short hypopneas with minimal desaturations, preserved nocturnal oxygen levels, and normal liver enzymes. The second patient suffers from prolonged obstructive apneas featuring repetitive oxygen drops below 80%, intense sympathetic drive, a reverse-dipping blood pressure pattern, severe insulin resistance, and elevated hepatic transaminases. Despite their identical AHI score of 30, their clinical trajectories are entirely different. The second patient faces a drastically higher risk of heart failure, stroke, and progressive liver fibrosis [5,10].

We observe this exact disconnect across metabolic pathways as well. Chronic nocturnal hypoxia has been shown experimentally to accelerate hepatic de novo lipogenesis and to worsen systemic insulin resistance, a sequence that offers a plausible explanation for the liver fat accumulation and metabolic deterioration observed in patients with both OSA and MASLD, although in human cohorts the relationship remains associative [7,8,15]. Global epidemiological data confirm that metabolic liver dysfunction tracks closely with insulin resistance, visceral obesity, and systemic vascular risk [15]. Yet the AHI cannot distinguish between a patient whose sleep breathing induces severe metabolic exhaustion and one who remains metabolically compensated. By treating all respiratory events as biologically equivalent, traditional diagnostic protocols obscure the true clinical picture. Moving forward, risk assessment requires metrics that capture hypoxic load, blood pressure dynamics, and metabolic stress rather than simple event counts. This diagnostic blind spot is illustrated in Figure 2, which compares two hypothetical patients with an identical AHI of 30 events/hour but markedly different hypoxic burden, ambulatory blood pressure profiles, and target-organ damage, underscoring why respiratory event frequency alone cannot capture individual cardiovascular risk.

None of this diminishes the diagnostic role of the AHI. It defines the presence of obstructive sleep apnea, determines eligibility for treatment in most healthcare systems, is reproducible and inexpensive to derive from any diagnostic study, and remains the entry criterion for virtually the entire interventional literature, including every trial cited in this review. The argument developed here is not that the AHI should be replaced, but that it answers a different question from the one this review is concerned with: it establishes whether obstructive sleep apnea is present and how frequently events occur, not how much biological injury those events produce in a given patient.

### 3.2. Emerging Physiological and Cardiovascular Measures

A terminological distinction is useful before these parameters are described, since the manuscript refers to three categories of variable that operate at different levels. Physiological measures are quantities derived from a diagnostic recording, such as the AHI, the oxygen desaturation index, T90, hypoxic burden and the parameters obtained from ambulatory blood pressure monitoring; they describe the intensity or pattern of the disturbance. Circulating biomarkers are molecular analytes measured in blood or other body fluids, such as high-sensitivity troponins, natriuretic peptides or inflammatory mediators, and are considered in Section 8. Clinical phenotypes are recognizable patterns of disease expression in a patient, such as resistant hypertension, nocturnal hypertension or hypertension-mediated target-organ damage, and are the subject of Section 4. The relationship between the three is directional: physiological measures and circulating biomarkers are the means by which clinical phenotypes are identified and quantified, and they are not themselves phenotypes.

Among the parameters proposed to overcome these limitations, hypoxic burden has received the most consistent support. Rather than counting discrete respiratory events, it quantifies the area under the oxygen saturation curve attributable to individual respiratory events. In the method in which it was originally described, each scored apnea or hypopnea is assigned a search window whose duration is derived from the ensemble-averaged desaturation curve of that individual recording, rather than from a fixed interval; within that window, the area between the pre-event baseline saturation and the measured saturation is integrated; the resulting areas are summed across all events and normalized to total sleep time, yielding a value expressed in percent-minutes per hour of sleep [5,16]. In a joint analysis of the Sleep Heart Health Study and the Osteoporotic Fractures in Men Study, hypoxic burden, unlike the AHI, predicted cardiovascular mortality independently of conventional risk factors [5], and a later analysis of the same cohorts linked it specifically to incident heart failure [10]. A 2024 review situates hypoxic burden within a broader family of area-based hypoxemia metrics and argues that this dimension of severity, largely invisible to the AHI, may account for much of the unexplained variance in cardiovascular outcomes among patients with comparable event counts [16].

The distinction from the two conventional hypoxemia indices is therefore computational as much as conceptual. The oxygen desaturation index is a rate: it counts, per hour of sleep, the desaturations exceeding a fixed threshold, most often three or four percent, and treats every qualifying event identically, so that a shallow three-percent dip and a prolonged ten-percent desaturation contribute equally while a two-percent dip contributes nothing. T90 is a duration: it sums the time spent below an absolute saturation of ninety percent, expressed in minutes or as a proportion of total sleep time, so that it registers how long a patient remains hypoxemic but neither how far below the threshold the saturation falls nor whether the hypoxemia is attributable to obstructive events at all, which is why it is inflated by baseline hypoxemia in patients with coexisting COPD, obesity hypoventilation or heart failure. Hypoxic burden is an integral: it is threshold-free, incorporates depth and duration jointly, and attributes the resulting area to individual respiratory events, which isolates the component of nocturnal hypoxemia specific to sleep-disordered breathing. This last property is also the source of its principal practical limitation, since the computation requires automated analysis of the oximetry signal synchronized with event scoring and is not implemented in routine clinical reporting.

Two qualifications should temper this picture, and they apply throughout the review. First, no consensus threshold for hypoxic burden currently exists. The cohorts cited above analyzed it as a continuous variable or by distribution-derived quantiles specific to each study population, so the values that separate low from high risk are not transferable between cohorts and cannot at present be applied to an individual patient. Second, the prognostic advantage of hypoxic burden over the AHI, although consistent for cardiovascular mortality and incident heart failure, is not uniform across every cardiovascular endpoint examined; in at least one direct comparison the AHI remained the stronger predictor of morning diastolic blood pressure (Section 4.5). Hypoxic burden should therefore be regarded as the most promising of the emerging metrics rather than as an established replacement for the AHI, and the interpretations offered throughout this review are qualified accordingly.

Two further indices refine this picture. The oxygen desaturation index (ODI) counts desaturation events per hour rather than respiratory events per hour, tracking intermittent hypoxia more directly than the AHI, even though the two measures remain closely correlated. Cumulative sleep time spent below 90% oxygen saturation (T90) instead captures the duration of hypoxic exposure across the whole night; patients with prolonged T90 accumulate substantial hypoxic stress even when individual events are brief, and this parameter has been associated with resistant hypertension and atrial fibrillation independently of AHI category [6,11].

It follows that these metrics are better regarded as complementary than as competing, since each captures a different dimension of the same disorder and each carries its own limitations. The AHI defines the presence of disease, determines eligibility for treatment in most healthcare systems, and remains the entry criterion for virtually the whole of the interventional literature, which makes it indispensable regardless of its prognostic performance. The ODI counts desaturation events and can be derived from overnight oximetry alone, which makes it the most readily available of the hypoxemia metrics in settings where full polysomnography is not accessible, at the cost of remaining closely correlated with the AHI and of saying nothing about the duration of each desaturation. T90 quantifies the total duration of hypoxic exposure, but it is not specific to sleep-disordered breathing: in patients with coexisting COPD, obesity hypoventilation or heart failure, baseline hypoxemia inflates T90 independently of obstructive events, so its interpretation depends on the comorbidity profile of the population studied. Hypoxic burden, by quantifying the area under the desaturation curve attributable to individual respiratory events, isolates the OSA-specific component of nocturnal hypoxemia more precisely than the others, but it requires signal processing that is not implemented in routine clinical reporting, is not currently available in most sleep laboratories, and lacks the standardized thresholds that would be needed to apply it to an individual patient. The practical implications of this last point for resource-constrained settings are considered in Section 9.

The relative prognostic value of these metrics should therefore be expected to vary with both the population and the outcome under consideration. The cohorts in which hypoxic burden has performed best consisted predominantly of older adults, and in the case of one of the two principal cohorts exclusively of men, so the extent to which the findings extend to women, to younger patients, and to populations with a different comorbidity structure is not established. The outcome examined matters equally: the advantage of hypoxic burden over the AHI is most consistent for cardiovascular mortality and incident heart failure, is mixed for blood pressure endpoints, and should not be assumed for outcomes such as daytime sleepiness or neurocognitive impairment, which relate more closely to sleep fragmentation and arousal burden than to hypoxemia. Comparative statements about these metrics are accordingly qualified by outcome throughout this review rather than made in general terms.

A separate mechanism operates through sleep fragmentation itself. Each arousal triggers a burst of sympathetic activity, and this activation does not necessarily resolve once breathing normalizes, heart rate variability studies show altered autonomic tone persisting well into wakefulness [11,17]. The same sympathetic and inflammatory pathway is already implicated in Section 3.1 in hepatic lipogenesis and insulin resistance [7,8,18] is one reason the AHI performs no better as a marker of metabolic injury than it does as a marker of cardiovascular injury.

The AHI is similarly silent on how blood pressure behaves over 24 h. It carries no information on nocturnal dipping status, isolated nocturnal hypertension, morning blood pressure surge, or short-term blood pressure variability, despite these patterns being among the strongest independent predictors of cardiovascular events in hypertensive populations and disproportionately common in untreated OSA [17,19]. Detecting them requires ambulatory blood pressure monitoring; office readings, and by extension the AHI itself, cannot substitute for it.

The same blind spot extends to structural damage. Left ventricular hypertrophy, arterial stiffness, carotid atherosclerosis, and early renal impairment have each been associated with OSA independently of AHI severity, reflecting the cumulative rather than momentary nature of cardiovascular injury [17,20]. This may explain why the blood-pressure benefit of CPAP is so inconsistent across trials: the SAVE trial found no reduction in cardiovascular events with CPAP among patients with established cardiovascular disease and moderate-to-severe OSA [21], while patients with resistant hypertension and good adherence often fare better, a discrepancy that is difficult to reconcile if the AHI alone is taken as the measure of disease severity. Taken together, these observations support treating the AHI as a diagnostic threshold rather than a prognostic scale, setting the stage for the phenotype-based framework developed in the sections that follow.

### 3.3. Intermittent Hypoxia as the Principal Pathophysiological Driver

A note on terminology is necessary before the mechanisms are described. The pathways set out below are supported by three distinct kinds of evidence, which we distinguish throughout: controlled exposure experiments in animal models, in which intermittent hypoxia can be applied and its consequences observed directly; mechanistic and interventional studies in humans, which are smaller and generally address intermediate endpoints; and observational cohorts, which establish association but not direction of effect. Where a mechanism has been demonstrated experimentally in animals but supported in humans only by association, we describe it as plausible or as consistent with the human data rather than as established. Causal language is reserved for the chemoreflex-mediated blood pressure response, for which controlled human exposure data and interventional evidence are available.

Behind these metrics lies a single unifying mechanism. Recurrent cycles of desaturation and reoxygenation stimulate the peripheral chemoreceptors of the carotid body, driving a sustained rise in sympathetic outflow; this chemoreflex-mediated activation, together with impaired baroreflex buffering, is now recognized as the principal route by which intermittent hypoxia raises blood pressure [22]. The same cyclical hypoxia-reoxygenation pattern activates hypoxia-inducible factor-1 (HIF-1) and NF-κB signaling, generating reactive oxygen species, systemic inflammation, and renin-angiotensin-aldosterone system activation, which together impair endothelial nitric oxide availability. Critically, this cascade does not switch off once breathing resumes: chemoreflex sensitization and sympathetic tone persist into wakefulness, which is why intermittent hypoxia, rather than the respiratory event itself, produces sustained daytime hypertension and cumulative target-organ injury rather than a transient nocturnal rise [17,22]. The distinction between the acute and the chronic chemoreflex response deserves particular emphasis, since the two are frequently conflated and carry different clinical implications. The acute response is stimulus-locked. During an obstructive event, falling arterial oxygen tension depolarizes the glomus cells of the carotid body, afferent traffic in the carotid sinus nerve increases, and the signal is relayed through the nucleus tractus solitarius to the rostral ventrolateral medulla, producing a sympathetic burst, peripheral vasoconstriction and a transient rise in arterial pressure that resolves with arousal and reoxygenation. Taken in isolation, this is a protective reflex operating as intended.

Repeated exposure, however, appears to alter the reflex itself. Experimental work in chronic intermittent hypoxia models has demonstrated sensitization of the carotid body, with augmented basal and hypoxia-evoked afferent discharge that outlasts the hypoxic stimulus. The mechanisms implicated include reactive oxygen species generated by NADPH oxidase, an imbalance between HIF-1α and HIF-2α signaling, local angiotensin II acting on AT1 receptors expressed by glomus cells, endothelin-1, and inflammatory cell recruitment within the carotid body itself. The functional consequence is a shift from an intermittent, stimulus-locked reflex to a tonically elevated sympathetic outflow that persists during normoxic wakefulness, accompanied by impaired baroreflex buffering and by increased renal sympathetic traffic favoring sodium retention. A recent synthesis of chemoreflex function across acute and chronic pulmonary disorders places the OSA phenotype within this broader framework and underscores that chemoreflex sensitization, rather than the respiratory event itself, is the more plausible substrate for the sustained cardiovascular consequences of the disease [23].

In humans, the supporting evidence is largely indirect but consistent in direction. Awake, normoxic patients with OSA show elevated muscle sympathetic nerve activity and exaggerated ventilatory and pressor responses to hypoxic challenge, both of which are attenuated, though not always normalized, by CPAP. Transient hyperoxic deactivation of the carotid body lowers sympathetic outflow and blood pressure in these patients, indicating that a tonic peripheral chemoreflex drive contributes measurably to their daytime hypertension. Consistent with the terminology adopted at the beginning of this section, the sensitization mechanism itself is established experimentally in animal models, while the human evidence establishes that the expected functional signature is present rather than that the mechanism operates identically in patients.

The same HIF-1–driven cascade extends beyond the vasculature. In the liver and adipose tissue, hypoxia-inducible signaling has been shown to upregulate lipogenic pathways and downregulate PPAR-γ activity, with hepatic triglyceride accumulation and reduced insulin sensitivity following, in experimental models, independently of body weight [7,24]. The corresponding evidence in humans is observational, and the extent to which this pathway operates at the intensities of hypoxia encountered in clinical OSA has not been established. This is why oxyhemoglobin desaturation indices correlate not only with blood pressure phenotype, but also with insulin resistance, dyslipidemia, and hepatic steatosis in patients with OSA [8,24], the same mechanistic thread introduced in Section 3.1, now traced to its molecular origin in intermittent hypoxia itself.

As already noted, the AHI cannot capture this cascade’s true magnitude: a patient with fewer but longer, deeper desaturations may sustain more chemoreflex and oxidative stress than one with numerous brief shallow events, even at an identical event count. This is precisely the physiological reasoning behind hypoxic burden (Section 3.2), and it is why blood pressure phenotype, rather than respiratory frequency, is the more direct readout of how much hypoxia-mediated injury a given patient has actually sustained, which is the focus of Section 4.

### 3.4. Ambulatory Blood Pressure Monitoring: Making the Hidden Phenotype Visible

The blood-pressure blind spot introduced above has a specific clinical face. Isolated nocturnal hypertension, an elevated nighttime pressure with an apparently normal daytime reading, is frequently the earliest detectable sign of OSA-related cardiovascular injury, appearing well before any office measurement turns abnormal [2,19]. Current hypertension guidelines now list non-dipping, reverse-dipping, masked hypertension, and an exaggerated morning surge among the phenotypes most consistently associated with stroke, heart failure, chronic kidney disease, and cardiovascular death, and both the 2023 ESH and 2024 ESC guidelines recommend out-of-office measurement as the preferred, not merely supplementary, method of diagnosis in this population [19,25].

The reason is prognostic, not just diagnostic. Nocturnal blood pressure correlates more closely than office readings with left ventricular hypertrophy, albuminuria, arterial stiffness, and incident cardiovascular events [17,19,25], precisely the target-organ markers introduced in Section 3.2. Twenty-four-hour ABPM should therefore be considered a routine, not exceptional, component of cardiovascular assessment in hypertensive patients with OSA. Having established why phenotype matters more than frequency, we now turn to what these phenotypes actually look like in practice. The relative cardiovascular prognostic value, strength of evidence, and remaining knowledge gaps of these conventional and emerging measures, namely the AHI, ODI, T90, hypoxic burden and 24-h ABPM, are compared systematically in Table 1.

The role of the AHI is evolving from a prognostic marker to a primarily diagnostic one, while cardiovascular phenotype, captured through hypoxic burden, ambulatory blood pressure monitoring, and target-organ assessment, is emerging as the principal determinant of long-term cardiovascular risk in hypertensive patients with OSA.

## 4. Cardiovascular Phenotypes of Hypertension in Obstructive Sleep Apnea

Hypertension associated with obstructive sleep apnea should no longer be treated as a single clinical entity. As the preceding sections have shown, patients with comparable AHI values can differ substantially in hypoxic burden, sympathetic and metabolic activation, ambulatory blood pressure pattern, and consequently in cardiovascular prognosis and treatment response [17,27]. This heterogeneity reflects the same interacting mechanisms described above, intermittent hypoxia, sympathetic overactivity, vascular and metabolic dysfunction, converging with individual susceptibility to produce markedly different clinical pictures. A recent narrative synthesis of a decade of OSA phenotyping research reached a similar conclusion from a different angle, showing that symptom-based phenotypes identify cardiovascular risk that AHI-based severity alone misses entirely [28]. A phenotype-oriented framework, rather than a purely respiratory classification, therefore appears better suited to cardiovascular risk stratification in this population.

Throughout this review, the term cardiovascular phenotype is used in the sense established in contemporary cardiovascular medicine, that is, a clinically observable and reproducible expression of disease that carries distinct prognostic and therapeutic implications, rather than in a strict biological or genotype-derived sense. The six entities discussed below are not equivalent in kind, and we distinguish three conceptual domains among them. The first comprises circadian and temporal blood pressure patterns identified on ambulatory monitoring, namely isolated nocturnal hypertension, non-dipping and reverse-dipping profiles, and an exaggerated morning surge. The second comprises categories defined by measurement discordance or by response to treatment, namely masked hypertension, in which office and out-of-office values diverge, and resistant hypertension, which is defined by inadequate blood pressure control despite multidrug therapy. The third comprises hypertension-mediated target-organ damage, which is defined by the structural and functional consequences of sustained pressure and hypoxic injury rather than by blood pressure behaviour itself. We include this last category deliberately, since in clinical practice the presence of target-organ damage reclassifies cardiovascular risk and alters management intensity irrespective of the measured blood pressure pattern, as reflected in the framework proposed in Section 6. Grouping these entities under a single heading is therefore intended to denote a shared level of clinical description and a shared underlying mechanistic substrate, not conceptual equivalence.

Two related terms recur alongside it and are used in this review with distinct meanings. Cardiovascular risk denotes the probability of a future cardiovascular event, as estimated by validated instruments or by the presence of established risk factors, and is therefore a quantitative and prospective notion. Cardiovascular phenotype denotes the observable pattern of disease expression in a given patient, as defined above, and is descriptive and cross-sectional. Where we refer to cardiovascular vulnerability, we mean the underlying susceptibility to cardiovascular injury of which the phenotype is the clinical expression and the risk estimate the quantitative consequence; the term is used only where this mechanistic sense is intended, and it does not denote a measurable quantity.

None of the six entities described below is novel in itself. Each is an established clinical category, defined in current hypertension guidelines and identifiable with standard ambulatory monitoring, and each has an existing literature independent of sleep medicine. What this review proposes is not a new taxonomy but a reorganization: assembling these established categories into a single set defined by their shared relationship to intermittent hypoxia, and treating that set rather than respiratory severity as the relevant axis of cardiovascular risk in patients with OSA.

This shift mirrors a broader movement in cardiovascular medicine. Rather than relying on a single diagnostic threshold, phenotyping increasingly integrates clinical characteristics, imaging, biomarkers, and prognosis into clinically actionable subgroups, an approach already established for heart failure, where phenomapping has reclassified a syndrome once defined solely by ejection fraction into biologically distinct subtypes with different vascular and metabolic drivers [29]. Applying the same logic to hypertensive OSA recognizes that comparable respiratory severity can still produce distinct cardiovascular phenotypes with correspondingly different therapeutic needs, and these phenotypes are examined individually in the sections that follow.

Framed this way, OSA is better understood not as a purely respiratory disorder but as a systemic cardiovascular stressor, one whose cardiovascular expression depends on age, obesity, metabolic status, genetic susceptibility, and the cumulative duration of exposure to nocturnal hypoxemia, layered onto the chemoreflex, sympathetic, oxidative, and endothelial pathways described in Section 3.3. The clinically relevant question is accordingly shifting from how severe the sleep apnea is to how severe the cardiovascular phenotype it has produced actually is, a reframing that sits at the core of precision cardiovascular medicine and is the one this review adopts for the phenotype-by-phenotype analysis that follows.

### 4.1. Resistant Hypertension Phenotype

Resistant hypertension is the cardiovascular phenotype most consistently linked to OSA. Current guidelines define it as blood pressure remaining above target despite three antihypertensive agents of different classes, including a diuretic or controlled blood pressure requiring four or more medications [25,30]. The dose-dependent relationship between sleep-disordered breathing and hypertension was first established in large prospective cohorts nearly three decades ago [31,32], and has since been extended to long-term cardiovascular outcomes, with untreated severe OSA carrying substantially higher cardiovascular mortality than CPAP-treated disease [33]. Among patients specifically referred to hypertension clinics, OSA prevalence exceeds 70%, rising further among those with truly refractory disease, making it one of the most common identifiable contributors to treatment-resistant blood pressure [2,34].

The mechanisms driving this phenotype build directly on those described in Section 3.3. Recurrent intermittent hypoxia sustains chemoreflex-mediated sympathetic activation well beyond the sleep period itself, while parallel activation of the renin-angiotensin–aldosterone system promotes sodium retention, vascular remodeling, endothelial dysfunction, and increased arterial stiffness [22,24,25]. Elevated aldosterone appears to act bidirectionally: beyond its hemodynamic effects, it may worsen upper-airway edema and collapsibility, aggravating OSA severity and creating a self-sustaining loop between resistant hypertension and sleep-disordered breathing [35].

Clinically, this phenotype clusters with obesity, metabolic syndrome, diabetes mellitus, increased arterial stiffness, left ventricular hypertrophy, and chronic kidney disease, alongside persistently poor blood pressure control despite multidrug therapy [17,28]. These patients also appear to derive the greatest antihypertensive benefit from CPAP: in the HIPARCO randomized trial, CPAP produced a significant reduction in 24-h blood pressure among patients with resistant hypertension and OSA, an effect most pronounced with good adherence, supporting systematic OSA screening in resistant hypertension clinics [36].

### 4.2. Nocturnal Hypertension Phenotype

Ambulatory blood pressure monitoring has fundamentally changed the understanding of hypertension in OSA. A substantial proportion of patients maintain normal office readings while nonetheless exhibiting isolated nocturnal hypertension, a phenotype increasingly recognized as a distinct contributor to cardiovascular risk [25,37]. Each respiratory event provokes a transient surge in sympathetic activity together with abrupt swings in intrathoracic pressure; when these events recur dozens of times per hour, the resulting blood pressure elevations accumulate across the night rather than resolving between events [22,37].

Unlike office readings, nocturnal blood pressure tracks the cardiovascular consequences of sleep-disordered breathing far more closely, and OSA-related hypertension is now recognized to carry its own distinct signature, nocturnal hypertension alongside resistant and variable blood-pressure patterns that office measurement alone cannot capture [1,19]. Because isolated nocturnal hypertension, by definition, coexists with a normal daytime reading, it routinely escapes detection without dedicated ambulatory monitoring, delaying recognition of both the hypertension itself and the underlying OSA [25,34].

The prevalence of nocturnal hypertension rises with OSA severity, but severity alone does not fully account for it: hypoxic burden, obesity, age, and pre-existing cardiovascular disease each contribute independently, consistent with the broader phenotyping evidence discussed earlier in Section 3.2 [17,28,34]. Nocturnal hypertension should therefore not be treated merely as a downstream consequence of a high AHI, but as a distinct cardiovascular phenotype in its own right, one that specifically requires ambulatory monitoring to identify [37].

### 4.3. Non-Dipping and Reverse-Dipping Phenotype

Blood pressure normally falls by 10–20% during sleep. Loss of this physiological decline defines the non-dipping phenotype, while nighttime pressure exceeding daytime values defines reverse dipping. Both patterns are common among patients with OSA and, independently of mean 24-h blood pressure, predict stroke, heart failure, chronic kidney disease, and cardiovascular death [26,38].

Persistent sympathetic activation, impaired baroreflex sensitivity, endothelial dysfunction, and recurrent nocturnal arousals disrupt the normal circadian regulation of blood pressure [17,22]. Intermittent hypoxia compounds these mechanisms further, increasing oxidative stress and reducing nitric oxide bioavailability in a way that blunts the vascular relaxation the sleeping cardiovascular system would otherwise undergo [22].

Reverse dippers, in particular, show consistently greater left ventricular mass than patients with a normal circadian blood pressure pattern, a difference confirmed across echocardiographic studies even after adjustment for office blood pressure [39]. A similar excess has been reported for arterial stiffness and carotid intima-media thickness, indicating that the circadian pattern itself, not only the absolute blood pressure level, carries independent prognostic weight [26,40]. Ambulatory blood pressure monitoring is therefore indispensable for identifying this high-risk phenotype, since neither the AHI nor office blood pressure can substitute for it [38].

### 4.4. Masked Hypertension Phenotype

Masked hypertension, normal office blood pressure with elevated ambulatory or home readings, is another clinically relevant phenotype frequently encountered in OSA [2]. Because routine office measurements remain within normal limits, the diagnosis is often delayed until cardiovascular complications have already appeared.

OSA contributes to this phenotype through recurrent nocturnal sympathetic activation that persists into daytime autonomic function, sustaining ambulatory blood pressure elevation despite reassuring office readings [17,22]. These patients frequently show early hypertension-mediated organ damage, underscoring the limits of office blood pressure as a screening tool on its own [2,17].

The clinical stakes are considerable: a 2024 meta-analysis pooling more than 15,000 participants found that masked hypertension carries a cardiovascular mortality risk (pooled HR 2.05) statistically indistinguishable from that of sustained hypertension (HR 2.42), and substantially higher than normotension [41]. Current evidence therefore supports ambulatory blood pressure monitoring in patients with suspected OSA even when office blood pressure appears normal [2,19,25].

### 4.5. Morning Blood Pressure Surge Phenotype

The transition from sleep to wakefulness is normally accompanied by a physiological rise in sympathetic activity and blood pressure. In OSA, this transition becomes exaggerated: persistent nocturnal sympathetic stimulation carries over into the waking hours before autonomic tone has fully recovered, amplifying what would otherwise be a modest morning rise [22]. An excessive morning blood pressure surge has been independently associated with stroke, myocardial infarction, sudden cardiac death, and destabilization of vulnerable atherosclerotic plaque [42].

Patients with a pronounced morning surge tend to show more severe nocturnal hypoxemia and more advanced arterial stiffness, and a large retrospective cohort has confirmed that OSA is independently associated with morning hypertension [43]. Whether the surge itself tracks cumulative hypoxic exposure more closely than the AHI is still being worked out: when hypoxic burden was tested head-to-head against AHI, ODI, and arousal index, it proved the stronger predictor of the rise in morning catecholamines and heart rate, whereas AHI remained the better predictor of morning diastolic pressure specifically [44]. This partial dissociation reinforces, once again, that respiratory event frequency alone cannot be relied upon to capture the full cardiovascular consequences of OSA.

### 4.6. Hypertension-Mediated Target-Organ Damage as a Risk-Defining Phenotype

Beyond its effects on blood pressure, OSA accelerates structural cardiovascular remodeling. Left ventricular hypertrophy, concentric remodeling, left atrial enlargement, impaired diastolic function, increased arterial stiffness, carotid atherosclerosis, chronic kidney disease, retinal microvascular abnormalities, and cognitive dysfunction are increasingly recognized as manifestations of hypertension-mediated organ damage in this population [17,20,44].

These alterations reflect chronic exposure to intermittent hypoxia, oxidative stress, inflammation, and neurohumoral activation rather than respiratory event frequency alone [22,44]. At the histological level, this hypoxia-driven remodeling has a direct correlate: in patients with hypertension, atrial fibrillation, coronary artery disease, and heart failure, reduced myocardial capillary density has been shown to track closely with the extent of diffuse myocardial fibrosis, consistent with local hypoxia-driven fibroblast activation as the underlying mechanism [45]. A patient with only moderate AHI but significant target-organ damage should therefore be regarded as carrying a high cardiovascular-risk phenotype, warranting comprehensive evaluation and aggressive risk-factor modification regardless of how the respiratory severity is classified.

### 4.7. Sex Differences in Presentation and in the Evidence Base

A phenotype-oriented framework should account for sex, both because the clinical expression of OSA differs between men and women and because the evidence underpinning the framework was generated largely in men. Women with OSA more often present with insomnia, fatigue, morning headache and mood symptoms than with witnessed apneas and habitual snoring, which contributes to delayed recognition and under-referral [46,47]. Polysomnographic expression also differs: the overall apnea-hypopnea index is lower, individual events are shorter, positional disease is less common, and events cluster in REM sleep, with AHI values during REM sleep that are essentially equivalent to those of men despite substantially lower non-REM values [47,48]. Hypopnea definitions requiring a four percent desaturation underestimate the index in women, and because treatment eligibility rests on the total AHI, which predominantly reflects non-REM events, women are correspondingly less likely to meet criteria for treatment [48]. Prevalence rises after the menopause, with a two- to threefold increase in the odds of sleep-disordered breathing independent of age, body habitus and smoking, narrowing but not eliminating the difference from men [49]. Taken together, these observations imply that AHI thresholds derived predominantly from male cohorts may misclassify cardiovascular risk in women, which is an argument for the phenotype-oriented approach advanced here rather than against it.

The same observation, however, applies to the evidence on which that approach rests, and this should be stated plainly. The cohorts that established the prognostic value of hypoxic burden were composed predominantly of older adults and, in the case of the Osteoporotic Fractures in Men Study, exclusively of men. The randomized trials of CPAP in secondary prevention likewise enrolled predominantly male populations. Whether the phenotypes described in this section carry the same prognostic weight in women, and whether the measures used to detect them perform equivalently, has not been established. Sex-stratified analysis should therefore be a requirement of any prospective evaluation of phenotype-guided management, and is not an optional refinement of it.

### 4.8. Toward Cardiovascular Phenotyping

Collectively, these observations indicate that hypertension associated with OSA encompasses multiple cardiovascular phenotypes, each with distinct mechanisms, prognostic implications, and therapeutic responses. Resistant hypertension, nocturnal hypertension, non-dipping or reverse-dipping blood pressure, masked hypertension, exaggerated morning blood pressure surge, and advanced hypertension-mediated target-organ damage are therefore best understood not as isolated clinical entities but as complementary expressions of the same underlying cardiovascular vulnerability although they are defined at different conceptual levels, as outlined at the beginning of this section [20,28].

Recognizing these phenotypes is a first step toward precision cardiovascular medicine in OSA. Rather than asking only how severe the sleep apnea is by AHI, clinicians are increasingly encouraged to ask which cardiovascular phenotype is present, which mechanisms are driving the blood pressure elevation, and which patients are most likely to benefit from a given intervention, a shift already reflected in recent treatment-focused reviews emphasizing that CPAP’s modest average effect on blood pressure conceals substantial heterogeneity in individual response [50]. This phenotype-oriented perspective forms the conceptual basis for the integrated cardiovascular assessment proposed in the following section. The defining features, principal mechanisms, and distinctive prognostic markers of the six cardiovascular phenotypes discussed above are summarized comparatively in Table 2.

Table 3 complements this comparison by setting out, for each phenotype, the nature of the supporting evidence, its reported prevalence in populations with OSA, its relationship with the apnea-hypopnea index and with hypoxic burden, and whether its identification alters clinical management.

## 5. Beyond Respiratory Indices: Integrating Cardiovascular and Metabolic Assessment

The growing recognition of cardiovascular heterogeneity in OSA has challenged the traditional assumption that respiratory severity alone should guide clinical decision-making. The AHI remains indispensable for diagnosis, but on its own, it says little about the cardiovascular or, as the preceding sections have shown, the metabolic consequences of sleep-disordered breathing. A growing body of evidence instead supports a multidimensional approach that pairs respiratory physiology with cardiovascular and metabolic evaluation, improving risk stratification and identifying which patients are most likely to benefit from targeted intervention [28,50,54].

A comprehensive assessment should begin with careful characterization of the hypertensive phenotype. Office blood pressure remains the cornerstone of hypertension diagnosis, but it incompletely captures the circadian abnormalities common in OSA. Ambulatory blood pressure monitoring provides substantially more prognostic information, identifying nocturnal hypertension, non-dipping or reverse-dipping patterns, exaggerated morning surge, masked hypertension, and increased blood-pressure variability, each independently associated with cardiovascular morbidity and mortality, and each therefore an essential component of cardiovascular evaluation in suspected or confirmed OSA [19,25,38,39].

The position of MASLD within this framework should be stated explicitly, since it is referred to at several points below. We treat MASLD primarily as a manifestation of the shared cardiometabolic phenotype rather than as an independent cardiovascular risk modifier in the sense in which hypertension-mediated target-organ damage is used in Section 4. The rationale is that the pathways implicated in hepatic steatosis in this population, namely intermittent hypoxia, sympathetic activation, insulin resistance and adipose tissue dysfunction, are the same pathways that produce the cardiovascular phenotypes described above, so that hepatic involvement serves as an accessible readout of a metabolic axis that is otherwise difficult to quantify at the bedside. MASLD is an established cardiovascular risk factor in the general population, and a mediating role between intermittent hypoxia and cardiovascular injury is mechanistically plausible; neither, however, has been demonstrated specifically in patients with OSA, and no evidence indicates that the presence of MASLD should alter cardiovascular management in this group beyond what its metabolic components already dictate. Its place in the framework is therefore diagnostic rather than therapeutic: it identifies patients in whom the metabolic axis is active, and it is on that basis alone that hepatic assessment is recommended in the sections that follow.

Because the same intermittent-hypoxia and sympathetic pathways that shape these blood-pressure phenotypes also contribute to insulin resistance and hepatic fat accumulation (Section 3.3), a comprehensive assessment should not stop at the cardiovascular axis. Repeated nocturnal hypoxic surges are associated with systemic insulin resistance and with markers of hepatic de novo lipogenesis, and the experimental data reviewed in Section 3.3 make a contribution to MASLD progression mechanistically plausible; whether treating OSA alters the course of MASLD in humans has not been demonstrated [15,24]. In these patients, high sympathetic drive impairs glucose handling and elevates nocturnal arterial pressure simultaneously [22,24]. Basic metabolic screening, fasting glucose or HbA1c, a lipid panel, and consideration of hepatic steatosis where clinically indicated, captures a dimension of risk that blood pressure phenotyping alone cannot, particularly given that the association between OSA and insulin resistance persists after adjustment for body weight in several cohorts [24,54]. Integrating this metabolic layer alongside ABPM-based phenotyping moves cardiovascular assessment in OSA closer to the multidimensional model this review has argued for throughout.

Beyond blood pressure tracking, evaluating hypertension-mediated organ damage gives us a clearer picture of true patient risk. Structural cardiac changes often appear long before clinical heart disease develops. These early shifts mark prolonged exposure to repetitive nocturnal hypoxemia and sympathetic overdrive. Transthoracic echocardiography serves as our primary imaging tool here. It measures left ventricular geometry, myocardial mass, diastolic relaxation, left atrial volume, and right heart pressures. Left ventricular hypertrophy, impaired diastolic filling, and left atrial enlargement represent the most common structural findings in hypertensive patients with OSA [44,51]. Notably, these cardiac changes have been reported to correlate more closely with cumulative hypoxic burden than with apnea counts [20,55], although the comparison derives from cross-sectional analyses.

We see a parallel acceleration of organ damage when metabolic dysfunction enters the picture. Chronic intermittent hypoxia and insulin resistance jointly increase myocardial fat deposition and arterial stiffness [24,29]. In patients who also present with MASLD and metabolic syndrome, hepatic inflammation and systemic oxidative stress amplify left ventricular diastolic dysfunction. This combined cardiometabolic strain worsens subclinical organ damage, reinforcing why we must evaluate cardiac structure alongside liver and metabolic health [7,24].

Evaluating the vascular tree gives us essential insights into structural arterial remodeling. By measuring carotid intima-media thickness, tracking carotid atherosclerotic plaques, checking pulse wave velocity, and recording central aortic pressure, we can spot early vascular damage before hard cardiac events occur [1,29]. Patients with obstructive sleep apnea often suffer from accelerated vascular aging. This process presents as heightened arterial stiffness, reduced compliance, and endothelial dysfunction [29].

Repeated nocturnal hypoxemia, systemic inflammation, and oxidative stress contribute to these vascular changes [34]. In parallel, insulin resistance and MASLD, both of which co-occur with OSA more often than chance would predict, appear to compound the vascular injury. Hepatic fat accumulation and circulating inflammatory lipids impair nitric oxide bioavailability, which stiffens the large arteries even faster. First, these combined factors elevate central blood pressure. Then, they sustain resistant hypertension over time [24].

An additional dimension that deserves increasing attention is autonomic function. Persistent sympathetic activation represents one of the principal mechanisms linking OSA with hypertension and cardiovascular disease, and four complementary approaches to its assessment should be distinguished, since they measure different things and differ substantially in availability.

Direct recording of muscle sympathetic nerve activity by microneurography remains the reference method for quantifying efferent sympathetic outflow. Patients with OSA show elevated burst frequency during normoxic wakefulness, which falls, though often incompletely, with effective CPAP therapy. The technique is technically demanding, available in few centers, and confined in practice to research use, but it is the standard against which indirect measures should be judged.

Heart rate variability is by far the most accessible of these measures, derivable from routine polysomnography and from consumer devices, and nocturnal heart rate profiles and resting heart rate can be obtained from the same recordings. Its interpretation, however, requires caution. High-frequency power reflects predominantly vagal modulation of the sinus node; low-frequency power has mixed autonomic and mechanical determinants; and the ratio of the two, long presented as an index of sympathovagal balance, does not correspond to any measurable physiological quantity and should not be reported as a measure of sympathetic activity. Heart rate variability characterizes cardiac autonomic modulation at the level of the sinus node, which is not equivalent to sympathetic outflow to the vasculature, and the two can diverge. In OSA specifically, respiratory events and arousals impose their own periodicity on the tachogram, so that nocturnal indices reflect the breathing disturbance as much as autonomic state.

Baroreflex sensitivity quantifies the gain of the reflex that buffers short-term blood pressure fluctuations and can be estimated non-invasively from spontaneous beat-to-beat blood pressure and interval sequences. It is reduced in OSA in proportion to disease severity, is associated with target-organ damage independently of mean blood pressure, and improves partially with CPAP. It is more demanding than heart rate variability but considerably more accessible than microneurography.

Chemoreflex sensitivity, finally, addresses the afferent limb of the pathway described in Section 3.3 and is assessed through the ventilatory and pressor responses to controlled hypoxic or hypercapnic challenge. Heightened peripheral chemoreflex gain has been documented in OSA and is mechanistically closest to the sensitization process that underlies sustained sympathetic activation, which makes it conceptually the most informative of the four; it is also the least standardized, with protocols differing between laboratories in a way that limits comparison across studies [23].

None of these measures is currently used in routine clinical decision-making, and we do not propose that they should be. They are included here because they illustrate the value of assessing functional cardiovascular responses rather than relying exclusively on structural abnormalities, and because the discrepancies between them are informative in their own right: a patient with preserved heart rate variability indices may nonetheless have elevated sympathetic outflow and reduced baroreflex gain [56,57].

Importantly, cardiovascular assessment should not be interpreted independently from respiratory physiology. Instead, respiratory and cardiovascular variables should be considered complementary dimensions of a single disease process. For example, a patient with moderate OSA, high hypoxic burden, resistant hypertension, reverse-dipping blood pressure profile, left ventricular hypertrophy, and chronic kidney disease represents a substantially different cardiovascular phenotype than another patient with identical AHI but preserved nocturnal oxygenation, controlled hypertension, and no evidence of target-organ damage. Traditional respiratory classification alone cannot distinguish between these two clinical scenarios despite their markedly different prognostic implications.

This multidimensional perspective reflects the transition from disease classification toward precision cardiovascular medicine. Rather than asking whether OSA is mild or severe according to respiratory indices, clinicians should identify the overall cardiovascular phenotype by integrating respiratory severity, nocturnal hypoxemia, ambulatory blood pressure characteristics, target-organ damage, metabolic dysfunction, vascular remodeling, and autonomic activation. Such an approach recognizes that cardiovascular injury results from the cumulative interaction between these variables rather than from respiratory event frequency alone [20,55]. Taken together, contemporary evidence strongly supports abandoning the traditional separation between respiratory medicine and cardiovascular medicine in the management of hypertensive patients with OSA. The future of cardiovascular care in sleep-disordered breathing will likely depend on integrated diagnostic models capable of simultaneously evaluating respiratory burden, cardiovascular phenotype, target-organ damage, and metabolic risk. Building upon this concept, the following section proposes a conceptual phenotype-oriented framework designed to facilitate individualized cardiovascular assessment and therapeutic decision-making.

## 6. A Proposed Conceptual Framework for Cardiovascular Phenotyping in Hypertensive Patients with Obstructive Sleep Apnea

The growing body of evidence discussed in the previous sections demonstrates that cardiovascular risk in patients with obstructive sleep apnea (OSA) cannot be adequately estimated by respiratory event frequency alone. Cardiovascular injury arises instead from the interaction between intermittent hypoxia, autonomic dysfunction, circadian blood pressure abnormalities, metabolic dysregulation, vascular remodeling, and hypertension-mediated target-organ damage. These observations support a transition from a disease-centered approach toward phenotype-oriented cardiovascular medicine, and on the basis of the currently available evidence we propose an integrated clinical framework designed to facilitate cardiovascular risk stratification in hypertensive patients with OSA [28,50].

Before the framework is described, its status should be stated explicitly. The classification proposed below is a conceptual synthesis of the evidence reviewed in the preceding sections, developed by the authors on the basis of pathophysiological reasoning and current guideline recommendations. It was not derived from a patient-level dataset, it was not generated by data-driven clustering or any other statistical class-discovery method, and it has not been calibrated or validated in an independent cohort. The three categories described here are therefore not risk strata in the sense in which that term is used for validated instruments such as SCORE2 or the Framingham risk equations, and they should not be used to generate quantitative risk estimates. Their intended purpose is to organize clinical reasoning by making explicit which cardiovascular and metabolic dimensions deserve assessment in a hypertensive patient with OSA, and by indicating how the intensity of evaluation and follow-up might reasonably be graded across that spectrum. Prospective validation against hard cardiovascular outcomes is a prerequisite before any such classification could be recommended for routine use, and we return to this point in the limitations.

Unlike previous diagnostic algorithms, which primarily classify respiratory severity, the proposed model focuses on identifying patients at the highest cardiovascular risk. The framework is intended neither to replace the apnea–hypopnea index (AHI) nor existing hypertension guidelines, but rather to complement current recommendations by integrating respiratory and cardiovascular information into a unified clinical pathway. Its overall structure, spanning clinical suspicion, respiratory and cardiovascular phenotyping, risk stratification, and dynamically reassessed management, is summarized in Figure 3 and developed in the paragraphs that follow.

The evaluation begins with the identification of hypertensive patients in whom clinically relevant OSA is more probable. At this initial stage, systematic attention should be directed toward blood pressure features that are characteristically associated with sleep-disordered breathing, namely resistant hypertension, nocturnal hypertension, a non-dipping or reverse-dipping profile, and an exaggerated morning blood pressure surge. These features should be considered alongside anthropometric and clinical markers of risk, including obesity defined by a body mass index of at least 30 kg/m^2^, increased neck circumference, and excessive daytime sleepiness, as well as alongside the cardiovascular and metabolic comorbidities that most frequently accompany untreated OSA, such as atrial fibrillation, heart failure, type 2 diabetes mellitus, and chronic kidney disease. Validated screening questionnaires, including the STOP-Bang, the Berlin Questionnaire and the NoSAS score, should be used as complementary tools, although in patients presenting with the cardiovascular red flags described above these clinical findings should prevail over questionnaire scores when the decision to proceed to objective sleep testing is made [17,58].

Once OSA is suspected or confirmed by polysomnography or home sleep apnea testing, respiratory evaluation should extend beyond conventional AHI classification. In addition to the AHI itself, the assessment should systematically record the hypoxic burden, the oxygen desaturation index, the mean and minimum nocturnal oxygen saturation, and T90, with T80 documented whenever the available equipment permits. The temporal characteristics of respiratory events, in particular event duration, should be reported together with the arousal index and with a description of sleep architecture, and the phenotypic variants that carry distinct therapeutic implications, namely REM-related and positional OSA, should be explicitly identified. The presence of central respiratory events should likewise be documented, since it modifies both prognosis and the choice of positive airway pressure modality. Taken together, these variables define the respiratory burden phenotype and provide a more comprehensive estimate of the physiological stress imposed on the cardiovascular system than respiratory event frequency alone [5,6,11].

Cardiovascular phenotyping represents the core component of the proposed model. Rather than focusing exclusively on blood pressure values, the assessment integrates five complementary domains. The first of these is the blood pressure phenotype, which is established by combining office measurements with twenty-four-hour ambulatory blood pressure monitoring, so that daytime and nighttime pressures can be examined separately and nocturnal hypertension, non-dipping and reverse dipping identified, together with the morning blood pressure surge and the overall degree of blood pressure variability. The second domain concerns hypertension-mediated target-organ damage and is necessarily multi-organ in scope. Cardiac evaluation should quantify left ventricular mass index and relative wall thickness, and should document left ventricular diastolic dysfunction, left atrial enlargement and pulmonary artery pressure. Vascular assessment rests on pulse wave velocity, carotid intima-media thickness, the presence of carotid plaques, and central aortic pressure. Renal involvement is characterized by the estimated glomerular filtration rate together with albuminuria expressed as the urinary albumin to creatinine ratio, and retinal examination should be added when clinically indicated. The third domain is the metabolic phenotype, which encompasses body mass index and waist circumference, glycemic status as reflected by HbA1c and fasting glucose, the lipid profile, liver function, serum uric acid, and the presence or absence of the metabolic syndrome. The fourth domain is functional and captures the autonomic and cardiorespiratory consequences of the disease through heart rate variability, resting heart rate, clinical or laboratory evidence of sympathetic overactivity, and exercise capacity. The fifth domain summarizes the broader clinical profile, comprising age, sex, smoking status, previous cardiovascular disease, the duration of hypertension, the current antihypertensive regimen, and, in treated patients, adherence to CPAP therapy. Collectively, these domains characterize the cardiovascular phenotype of the individual patient, which reflects long-term cardiovascular risk more accurately than respiratory severity considered in isolation [20,28,29].

On the basis of this assessment, patients may be conceptually grouped into one of three cardiovascular risk categories. Patients with a low cardiovascular phenotype present with controlled hypertension and a normal ambulatory blood pressure profile, a low hypoxic burden, no demonstrable target-organ damage, preserved renal function and a favorable metabolic profile, and for them routine cardiovascular follow-up is appropriate. The intermediate cardiovascular phenotype is defined by a moderate hypoxic burden and a non-dipping profile, accompanied by mild left ventricular hypertrophy, early renal dysfunction, obesity or diabetes. These patients warrant closer cardiovascular surveillance, optimization of antihypertensive treatment, early initiation of CPAP, and an aggressive approach to weight reduction. The high cardiovascular phenotype comprises patients with resistant hypertension, severe nocturnal hypertension and reverse dipping, in whom a high hypoxic burden coexists with advanced left ventricular hypertrophy, chronic kidney disease, atrial fibrillation, heart failure or multiple metabolic abnormalities. Their management should be multidisciplinary and should involve cardiology, sleep medicine, nephrology, nutrition and primary care, and it is this group that probably derives the greatest cardiovascular benefit from integrated treatment strategies [34,50].

The proposed framework therefore shifts the guiding clinical question from how severe the sleep apnea is to how severe the cardiovascular phenotype is. This conceptual transition carries several potential advantages. It should improve the identification of patients at highest cardiovascular risk and refine the selection of candidates most likely to benefit from CPAP, while allowing antihypertensive therapy to be optimized according to the cardiovascular phenotype rather than according to respiratory severity. It should also promote earlier detection of hypertension-mediated organ damage and facilitate the recognition of patients with concurrent metabolic and hepatic vulnerability, including insulin resistance and MASLD, who may benefit from combined cardiometabolic management. Finally, it offers a means of integrating cardiology and sleep medicine within a single precision medicine model.

Unlike previous approaches centered exclusively on respiratory indices, the present framework acknowledges that cardiovascular prognosis is determined by the cumulative interaction between respiratory burden, nocturnal hypoxemia, blood pressure phenotype, metabolic and hepatic dysfunction, and target-organ injury. Cardiovascular phenotyping may accordingly represent the next step toward the personalized management of hypertensive patients with OSA [24,54].

## 7. Personalized Therapeutic Strategies According to Cardiovascular Phenotype

The management of hypertensive patients with obstructive sleep apnea has traditionally rested on two largely independent objectives: treating respiratory events with CPAP and pharmacologically lowering blood pressure. The growing recognition of cardiovascular heterogeneity among patients with OSA suggests that this one-size-fits-all approach is no longer adequate. Contemporary evidence instead supports individualized management based on cardiovascular phenotype rather than respiratory severity alone: patients with identical AHI values may require substantially different therapeutic strategies depending on their cardiovascular and metabolic profile [17,20,28,50]. A patient with resistant hypertension and a high hypoxic burden, for example, is unlikely to respond to the same combination of CPAP and antihypertensive therapy as a patient with well-controlled blood pressure at an identical AHI [24,54]. Figure 4 summarizes the graduation of therapeutic intensity proposed here across six management domains, namely OSA-specific therapy, blood pressure control, metabolic management, organ protection, lifestyle intervention, and follow-up, as detailed in the phenotype-specific sections below. This graduation is framework-derived; the interventions it distributes are not.

A distinction should be drawn at the outset between the two kinds of statement made in this section. The individual therapeutic components described below, namely antihypertensive drug selection, CPAP prescription and adherence optimization, management of obesity, diabetes, dyslipidemia and chronic kidney disease, the use of SGLT2 inhibitors and GLP-1 receptor agonists where metabolically indicated, anticoagulation and rhythm management in atrial fibrillation, and guideline-directed medical therapy in heart failure, are all derived from current hypertension, sleep-medicine, diabetes and cardiovascular guidelines and from the trial evidence cited alongside them. What is proposed here, and what has not been prospectively validated, is the allocation of these established interventions across the three cardiovascular risk categories and the corresponding graduation of therapeutic and follow-up intensity. Statements of the latter kind are identified below as framework-derived. They are intended to structure clinical reasoning and should not be read as recommendations carrying the same evidentiary standing as the guideline-based components they organize, nor as grounds for departing from current guideline recommendations in an individual patient.Green, amber, and red shading denote the low-, intermediate-, and high-risk phenotypes, respectively.

### 7.1. Low Cardiovascular-Risk Phenotype

Patients classified as having a low cardiovascular-risk phenotype typically exhibit controlled hypertension, preserved nocturnal blood pressure decline, limited nocturnal hypoxemia, absence of hypertension-mediated target-organ damage, and a favorable metabolic status characterized by normal glucose tolerance, no significant insulin resistance, and no evidence of hepatic steatosis. Although respiratory disturbances may be moderate or even severe according to AHI criteria, cardiovascular and metabolic remodeling remains minimal [5,17,24].

In this population, therapeutic priorities rest primarily on the optimization of lifestyle interventions. Weight reduction should be pursued when appropriate, together with regular physical activity, smoking cessation and moderation of alcohol consumption. Dyslipidemia and diabetes should be treated according to current recommendations, as should early hepatic steatosis where this is present. CPAP therapy retains a clear role in these patients, but its indication is driven principally by symptomatic benefit and by the prevention of disease progression rather than by an expectation of substantial short-term cardiovascular gain.

Because cardiovascular risk is relatively limited, follow-up should focus on the periodic reassessment of blood pressure, body weight, metabolic and hepatic profile, and CPAP adherence, since a shift toward insulin resistance or emerging hepatic steatosis may signal early metabolic drift even before cardiovascular markers change. Repeat ambulatory blood pressure monitoring (ABPM) should be considered if new cardiovascular risk factors develop or if symptoms worsen [2,17,24].

### 7.2. Intermediate Cardiovascular-Risk Phenotype

Patients with intermediate cardiovascular risk generally demonstrate moderate nocturnal hypoxemia, a non-dipping blood pressure profile, early left ventricular remodeling, obesity and insulin resistance, often with early or unrecognized hepatic steatosis or mild chronic kidney disease. This phenotype represents an important therapeutic window during which aggressive intervention may prevent progression toward irreversible cardiovascular and metabolic injury.

Management in this group rests on three interdependent pillars, the first of which is the optimization of antihypertensive therapy. Treatment should follow current hypertension guidelines while taking into account the pathophysiological mechanisms associated with OSA, and the preferred therapeutic combinations comprise angiotensin-converting enzyme inhibitors or angiotensin receptor blockers, long-acting calcium-channel blockers and thiazide-like diuretics, with mineralocorticoid receptor antagonists added when clinically indicated. Although no antihypertensive class has demonstrated clear superiority in OSA, suppression of sympathetic activity and adequate nocturnal blood pressure control appear particularly important [1,22]. In patients with coexisting insulin resistance, type 2 diabetes or MASLD, agents with established cardiometabolic benefit such as SGLT2 inhibitors or GLP-1 receptor agonists may offer a rational adjunct, addressing the metabolic and cardiovascular dimensions of this phenotype simultaneously [24,54].

The second pillar is CPAP therapy, for which adequate adherence remains fundamental. The evidence supporting it, however, is not uniform across outcome levels, and the four should be distinguished.

The effect on respiratory events and oxygenation is the largest and most consistent: effective CPAP abolishes obstructive events and nocturnal desaturations in the great majority of adherent patients, and this effect is reproducible across trials [17,45]. The effect on blood pressure is real but modest on average, in the range of a few millimetres of mercury on twenty-four-hour monitoring in unselected populations, and is substantially larger in the subgroups considered in this review, particularly in resistant hypertension, where the HIPARCO trial demonstrated a significant reduction in twenty-four-hour blood pressure that was most pronounced among adherent patients [36]. The effect on intermediate cardiovascular markers is more heterogeneous: reductions in circulating inflammatory markers, including CRP, IL-6 and TNF-α, have been demonstrated in meta-analysis of randomized trials [59], whereas effects on sympathetic activity, endothelial function and left ventricular mass have been reported with considerable variation in magnitude and across the populations studied.

The effect on major cardiovascular events is the level at which the evidence is weakest, and this should be stated plainly. The three randomized trials designed to test this question in secondary prevention, namely SAVE in patients with established cardiovascular or cerebrovascular disease, RICCADSA in patients with coronary artery disease, and ISAACC in patients with acute coronary syndrome, all reported neutral findings for major cardiovascular events in their primary intention-to-treat analyses [21,60,61]. All three enrolled patients with moderate-to-severe OSA on the basis of respiratory indices and excluded those with severe daytime sleepiness, and mean adherence was low in ISAACC in particular, at under three hours per night.

A post hoc analysis of the ISAACC trial addressed this question directly, stratifying participants by baseline hypoxic burden rather than by the AHI. A significant interaction was found between treatment arm and hypoxic burden category: among patients in the high hypoxic burden group, CPAP was associated with a reduction in the incidence of cardiovascular events (HR 0.57, 95% CI 0.34–0.96), whereas no such benefit was apparent at low hypoxic burden [62]. This is the closest existing test of the proposition advanced in this review, and it points in the expected direction; it is nonetheless a secondary analysis of a single neutral trial, conducted in non-sleepy patients following an acute coronary syndrome, and it establishes a hypothesis rather than confirming one. Observations that adherent patients fare better derive more generally from on-treatment and post hoc analyses, which cannot exclude confounding by the characteristics that make patients adherent in the first place [63]. The proposition that the neutral trial results reflect enrolment based on the AHI, and on populations with low symptom burden and limited hypoxic exposure, is a reasonable interpretation and one that motivates the framework advanced here [20]; it is nonetheless an interpretation of negative trials rather than positive evidence for an alternative selection strategy.

It follows that whether selecting patients for CPAP on the basis of cardiovascular phenotype improves hard cardiovascular outcomes remains unknown. No trial has enrolled patients on that basis, although analyses of individualized treatment effects in existing cohorts suggest that identifiable subgroups may respond differently [64,65]. The argument developed in this review is that such a trial is worth conducting, not that its result can be anticipated. Beyond these direct effects, improvements in daytime sleepiness frequently increase adherence to lifestyle modification and to antihypertensive treatment, so that some of the clinical benefit of CPAP is indirect.

The third pillar is weight reduction. Its efficacy in reducing upper airway collapsibility, hepatic fat and blood pressure is well established; the proposal that it be treated as a disease-modifying rather than adjunctive intervention specifically at this stage of the cardiovascular spectrum is framework-derived, and reflects the view that this is the point at which structural change remains reversible. Reduction of visceral adiposity decreases upper airway collapsibility and thereby lowers the hypoxic burden, improves insulin sensitivity and reduces hepatic fat content, attenuates systemic inflammation, and contributes to better blood pressure control. Combined dietary intervention, structured exercise and behavioral counseling provide superior long-term outcomes compared with isolated interventions, particularly in patients with coexisting insulin resistance or MASLD, where weight loss directly targets the shared metabolic driver of both conditions [24,54].

### 7.3. High Cardiovascular-Risk Phenotype

Patients in the high cardiovascular-risk phenotype are the primary target for integrated cardiovascular management. They typically present with resistant hypertension, severe nocturnal hypertension and reverse dipping, together with a marked hypoxic burden, advanced left ventricular hypertrophy, atrial fibrillation, chronic kidney disease, heart failure, diabetes mellitus and obesity. At this stage, management can no longer center on respiratory events alone and must address the cardiovascular phenotype as a whole, across several concurrent domains of intervention.

Blood pressure control must be intensive and repeatedly verified. Serial ABPM should guide therapeutic adjustments, with particular attention to nighttime blood pressure, the morning blood pressure surge, and overall blood pressure variability. Chronotherapy may be considered in selected patients, although recent randomized trials have left its benefit unsettled.

CPAP therapy requires optimization rather than simple prescription, since at this level of risk the act of prescribing alone is not sufficient. Clinicians should ensure individualized mask fitting, ongoing adherence monitoring, assessment of the residual AHI, correction of mask leaks, and active management of treatment intolerance, since suboptimal adherence substantially reduces the cardiovascular benefit that CPAP can offer.

Cardiometabolic optimization becomes essential in parallel, through aggressive management of obesity, diabetes, dyslipidemia and chronic kidney disease, and it is here that the metabolic and respiratory tracks of care converge most directly. Many patients in this phenotype also carry unrecognized insulin resistance or MASLD, which amplifies both hepatic and cardiovascular injury. Systematic screening for these conditions in patients with OSA is not currently mandated by sleep-medicine guidelines, and its recommendation here is framework-derived, following from the mechanistic overlap developed throughout this review. Current cardiovascular and diabetes guidelines now support GLP-1 receptor agonists and SGLT2 inhibitors, when indicated, as more than glucose-lowering agents, since they appear to offer additional cardiovascular protection through weight reduction, improved endothelial function and reduced systemic inflammation, benefits that plausibly extend to the shared metabolic burden of insulin resistance and hepatic steatosis as well [54,66,67,68].

Arrhythmia management deserves specific consideration, given that OSA meaningfully increases the risk of atrial fibrillation recurrence after catheter ablation [69]. Rhythm control strategies should therefore be paired with systematic OSA treatment, anticoagulation should follow current guideline recommendations, and long-term rhythm monitoring may be worth considering in selected high-risk patients.

In patients with heart failure, guideline-directed medical therapy remains the foundation of care. Treatment of OSA can improve nocturnal oxygenation, sympathetic activity, sleep quality and quality of life, but its effect on hard cardiovascular outcomes is still under investigation, which reinforces the view that treatment decisions in this group need to remain individualized rather than protocol-driven [17].

### 7.4. Multidisciplinary Care

One of the main limitations of current clinical practice is the fragmentation between specialties. Optimal management of this population requires collaboration among cardiologists, sleep physicians and hypertension specialists, working alongside nephrologists, endocrinologists and, particularly when MASLD or significant insulin resistance is present, hepatologists, with nutritionists and primary care physicians completing the team. This multidisciplinary model allows respiratory disease, hypertension, metabolic and hepatic dysfunction, and target-organ damage to be addressed simultaneously rather than sequentially, which in turn tends to improve therapeutic adherence and long-term cardiovascular prevention [28,50].

### 7.5. Dynamic Cardiovascular Phenotyping

An important feature of the proposed model is that it is dynamic rather than static. Cardiovascular phenotype should not be regarded as fixed at the time of diagnosis and should instead be periodically reassessed as the clinical picture evolves.

Not all components of the phenotype are equally modifiable, and the purpose of reassessment differs accordingly. Three groups may be distinguished. The first comprises measures that respond within weeks to months to effective treatment: nocturnal hypoxemia, whether expressed as hypoxic burden, the oxygen desaturation index or T90, together with nocturnal blood pressure, dipping status, the morning surge, indices of sympathetic activation, glycemic parameters and hepatic fat content. These are the appropriate targets of short-interval reassessment, since a change in them constitutes evidence that treatment is working. The second comprises structural abnormalities that regress slowly and incompletely over months to years, including left ventricular mass, diastolic function, arterial stiffness and albuminuria; regression of left ventricular mass is well documented with effective antihypertensive treatment, whereas the evidence that treating OSA independently contributes to it is more limited and inconsistent; in either case the magnitude is modest and the time course long, so reassessment at intervals shorter than a year is unlikely to be informative [70]. The third comprises abnormalities that should be regarded as fixed for the purposes of this framework, including established coronary atherosclerosis, myocardial scar, permanent atrial fibrillation, advanced hepatic fibrosis and reduced glomerular filtration rate attributable to established chronic kidney disease. These are documented once and thereafter anchor the patient’s risk category rather than serving as endpoints of treatment; their presence continues to justify the higher intensity of management described above even if every modifiable component has normalized.

This distinction has a practical consequence for what dynamic phenotyping can and cannot demonstrate. Improvement in the first group indicates that the intervention has altered the physiological exposure; it does not by itself indicate that cardiovascular risk has fallen, since that inference depends on the outcome trials discussed in Section 7.2 and remains unproven. Conversely, the absence of change in the third group is not evidence of treatment failure.

Follow-up may accordingly be organized around three levels of intensity, corresponding broadly to the three groups above. The intervals proposed below are framework-derived and pragmatic rather than evidence-based. No trial has compared follow-up schedules in this population, and current hypertension and sleep-medicine guidelines specify when ambulatory blood pressure monitoring and echocardiography are indicated but not at what interval they should be repeated in patients with OSA. The intervals are therefore our own, derived from the rate at which the abnormalities concerned are expected to change and from what is practicable in routine care, and are offered as a starting point for local adaptation rather than as recommendations. At intervals of six to twelve months, reassessment should include office blood pressure, body weight, CPAP adherence, and laboratory evaluation comprising glycemic and hepatic panels. At intervals of twelve to twenty-four months, this core assessment should be extended to include ambulatory blood pressure monitoring, echocardiography, renal evaluation and assessment of vascular damage. Beyond this schedule, repeat polysomnography, cardiac rhythm monitoring, coronary evaluation and reassessment of heart failure status should be performed whenever clinically indicated rather than at predetermined intervals.

Dynamic phenotyping recognizes that cardiovascular and metabolic risk evolves over time in response to aging, therapeutic interventions, weight changes, glycemic and hepatic status, and the progression of target-organ damage. Therapeutic strategies should therefore evolve as well, rather than remaining fixed after the initial diagnosis [28,29,50].

### 7.6. Future Directions for Personalized Cardiovascular Therapy

The integration of respiratory physiology, ambulatory blood pressure monitoring, multimodal imaging, biomarkers, and artificial intelligence is likely to redefine cardiovascular management in OSA over the coming decade. Machine learning algorithms capable of simultaneously analyzing hypoxic burden, blood pressure phenotypes, cardiac remodeling, metabolic and hepatic profile, and autonomic function may enable individualized prediction of cardiovascular outcomes and facilitate precision therapeutic decision-making [64]. This is no longer purely theoretical: a causal machine learning model applied to data from the SAVE trial has recently demonstrated that individualized treatment-effect scores can identify which OSA patients are likely to benefit from CPAP cardiovascularly and which are not a concrete early example of the phenotype-driven precision approach this framework anticipates [65]. Although prospective validation remains necessary, phenotype-guided management, extended to the metabolic and hepatic dimensions discussed throughout this review, represents a promising strategy to overcome the limitations of current AHI-based approaches and to optimize cardiovascular prevention in hypertensive patients with OSA. The correspondence between cardiovascular risk phenotype, primary therapeutic focus, and recommended follow-up intensity, as detailed in Section 7.1, Section 7.2 and Section 7.3, is summarized in Table 4. Phenotype assignment should be reassessed dynamically rather than fixed at diagnosis (Section 7.5), as patients may transition between categories with treatment, weight change, or disease progression.

## 8. Future Perspectives: From Respiratory Disease to Precision Cardiovascular Medicine

OSA management is undergoing a paradigm shift. Historically viewed as a respiratory disorder defined almost exclusively by the AHI, the condition is now understood in the light of contemporary evidence as the expression of a far more complex interaction between nocturnal hypoxemia, autonomic dysfunction, circadian blood pressure abnormalities, vascular remodeling, insulin resistance and hepatic dysfunction, and hypertension-mediated target-organ damage, and it is this interaction rather than respiratory event frequency that determines cardiovascular prognosis. This understanding underlies the transition toward precision cardiovascular medicine in OSA [4,5,11,20], in which single-parameter respiratory classification gives way to multidimensional phenotyping integrating physiological, imaging, biochemical and clinical data.

Several converging developments are likely to shape this transition. The first is digital medicine and continuous monitoring, since wearable technologies now allow continuous tracking of heart rate, nocturnal oxygen saturation, heart rate variability, respiratory rate and sleep architecture. Pairing these with ambulatory blood pressure monitoring and, where relevant, with continuous glucose monitoring may enable longitudinal assessment of cardiovascular and metabolic burden in real-world settings, capturing treatment adherence and early deterioration well before a single-night polysomnogram would.

Artificial intelligence and predictive phenotyping represent a second development. Machine-learning algorithms can simultaneously analyze respiratory indices, hypoxemia, blood pressure profiles, imaging, laboratory and metabolic biomarkers, and clinical characteristics, potentially revealing phenotype interactions that traditional statistics miss. This is already moving from concept to practice. A recent narrative review outlined the framework [64], and a causal machine learning model applied to SAVE trial data has since produced individualized predictions of which OSA patients derive cardiovascular benefit from CPAP and which do not [65], work that builds on the broader convergence of AI and medicine described by Topol [72]. Extending such models to predict metabolic and hepatic, and not only cardiovascular, treatment response is a natural next step, although prospective validation remains necessary before clinical implementation.

Emerging biomarkers constitute a third avenue. Circulating markers currently under investigation include high-sensitivity cardiac troponins, NT-proBNP, high-sensitivity CRP, IL-6 and TNF-α, together with markers of oxidative stress, endothelial microparticles, circulating microRNAs, and hepatic markers such as FIB-4 or cytokeratin-18 fragments reflecting steatohepatitis activity. None of these yet supports routine clinical use, but combined cardiac and metabolic panels may eventually improve early detection and the dynamic monitoring of treatment response. Gut microbiota composition is emerging as a further dimension of metabolic risk in this context, since dysbiosis has been increasingly implicated in insulin-resistance-related disorders, which suggests that microbiome-targeted strategies may eventually complement the biomarker and imaging approaches described above [73].

Multimodal imaging offers a fourth line of development. Cardiac MRI, speckle-tracking echocardiography, coronary CT angiography, vascular MRI, myocardial strain analysis and epicardial adipose tissue quantification are increasingly sensitive to subclinical remodeling that is often detectable before ejection fraction declines. Paired with hepatic fat quantification, for example by MRI-PDFF, where MASLD is suspected, multimodal imaging could anchor a genuinely combined cardiohepatic phenotyping algorithm.

Within this avenue, extracellular vesicles warrant particular attention as a distinct class of candidate markers. Released by most cell types and carrying proteins, lipids and nucleic acids to recipient cells, they function as mediators of intercellular communication rather than as passive by-products of tissue injury, and their cargo reflects the state of the cell of origin. This dual character is what makes them conceptually attractive here: endothelial-, platelet-, leukocyte- and adipocyte-derived vesicles could in principle report simultaneously on the four processes that this review treats as the mechanistic substrate of cardiovascular risk in OSA, namely endothelial dysfunction, systemic inflammation, subclinical tissue injury and cardiometabolic remodeling, and they are accessible through peripheral blood sampling. Their potential is therefore that of a molecular counterpart to the physiological phenotyping described throughout this review, capable in principle of indicating which mechanisms are active in a given patient rather than only which functional abnormalities have already appeared. Considerable obstacles remain before any clinical application can be envisaged. Standardization of isolation and characterization methods is still lacking, attributing circulating vesicles to a specific tissue of origin is technically difficult, and prospective data in hypertensive patients with OSA are essentially absent; scalability and manufacturing constraints are further recognized as limitations for any translational use. We include this line of work as a conceptual direction rather than as an established or emerging clinical tool [74].

Precision therapeutics form a fifth dimension. Future treatment is likely to move beyond universal CPAP prescription, with decisions increasingly guided by cardiovascular phenotype, hypoxic burden, autonomic profile, inflammatory activity, metabolic and hepatic phenotype, obesity severity, genetic susceptibility and expected adherence. Combined interventions bringing together CPAP, weight management, structured exercise, cardioprotective pharmacotherapy and metabolic optimization are likely to matter most for patients with advanced cardiovascular and metabolic phenotypes alike.

Several research priorities follow from this agenda. Key open questions include whether cardiovascular phenotype predicts long-term CPAP response, which biomarkers best identify the highest-risk patients, and whether phenotype-guided treatment outperforms conventional management. Equally unresolved are the role of hypoxic burden as a therapeutic target, the manner in which ABPM should be integrated into routine OSA evaluation, the clinical utility of AI-assisted phenotyping, and whether treating insulin resistance or MASLD alongside OSA meaningfully changes cardiovascular outcomes. Randomized trials designed around cardiovascular phenotype rather than respiratory severity may be the next major step.

Taken together, these developments point toward a future in which hypertensive patients with OSA are characterized not by a single respiratory index but by the full extent of their cardiovascular and metabolic vulnerability. The AHI will remain essential for diagnosis, but as one component among many, alongside ambulatory blood pressure monitoring, multimodal imaging, metabolic and hepatic evaluation, biomarkers and artificial intelligence, within a precision medicine framework in which cardiovascular phenotype rather than respiratory severity alone ultimately guides prognosis and therapy.

## 9. Obstructive Sleep Apnea, Cardiovascular Risk, and Multimorbidity in Low- and Middle-Income Countries

The cardiovascular consequences of obstructive sleep apnea may be particularly important in LMICs, where the epidemiological transition has produced a rapidly increasing burden of obesity, hypertension, type 2 diabetes mellitus, chronic kidney disease, and cardiovascular disease [75]. These conditions frequently coexist in the same patient and overlap mechanistically with OSA through visceral adiposity, insulin resistance, sympathetic activation, endothelial dysfunction, systemic inflammation, and oxidative stress. Consequently, OSA in LMICs should not be considered exclusively as a sleep disorder, but rather as a component of a broader cardiometabolic multimorbidity phenotype [76,77].

The relevance of this to the framework proposed here is that cardiometabolic multimorbidity concentrates precisely those combinations that the framework is designed to identify, so that the discriminative value of phenotyping should be greatest where diagnostic resources are scarcest [45,77,78,79]. That is the tension this section addresses. At the same time, implementation of comprehensive OSA phenotyping in LMICs faces important structural barriers. Access to polysomnography, sleep specialists, ambulatory blood-pressure monitoring, advanced cardiovascular imaging, and CPAP therapy may be limited or concentrated in urban tertiary centers. Underdiagnosis is therefore likely to disproportionately affect patients with asymptomatic or minimally symptomatic OSA, masked or nocturnal hypertension, and cardiometabolic multimorbidity. Reliance on conventional symptom-based screening may be particularly problematic because individuals at high cardiovascular risk do not invariably report excessive daytime sleepiness. In LMICs, COPD should also be considered an important modifier of cardiovascular risk. OSA–COPD overlap may produce more severe nocturnal hypoxemia and greater cardiopulmonary stress than either disorder alone, particularly in populations exposed to smoking, biomass fuels, or air pollution. Incorporating respiratory symptoms, exposure history, and simple spirometry where feasible may therefore strengthen resource-adapted cardiovascular phenotyping [80].

A pragmatic LMIC-oriented strategy should emphasize scalable, resource-adapted cardiovascular risk assessment. Clinical screening combining obesity, resistant hypertension, diabetes, chronic kidney disease, atrial fibrillation, heart failure, and established cardiovascular disease with validated OSA questionnaires and nocturnal oximetry may help identify high-risk patients. Home sleep apnea testing and selective use of ABPM could provide practical alternatives when polysomnography and universal ambulatory monitoring are not feasible [77,78].

Implementation of emerging metrics such as hypoxic burden remains challenging because of the need for appropriate signal acquisition and analytical infrastructure. Standardized, automated methods deriving hypoxic burden from portable sleep studies or wearable devices could facilitate wider use in resource-constrained settings.

Cardiovascular phenotyping in LMICs should follow a tiered approach: basic clinical and metabolic assessment in primary care, portable sleep testing and ABPM in higher-risk patients, and advanced cardiac or vascular evaluation in those with suspected target-organ damage. Importantly, LMIC populations remain underrepresented in OSA cohorts and cardiovascular trials; prospective studies are needed to determine whether phenotype-based risk models developed predominantly in high-income countries are equally applicable across diverse populations.

## 10. Discussion and Conclusions

Obstructive sleep apnea and arterial hypertension form one of the most consequential intersections in contemporary cardiovascular medicine, yet the field has continued to lean on a single number, the AHI, to capture a problem that is anything but one-dimensional. As this review has shown, two patients with identical AHI values can differ substantially in nocturnal hypoxemia, sympathetic activation, ambulatory blood pressure behavior, and target-organ damage, and consequently in long-term cardiovascular risk [5,11,20]. Respiratory event frequency, in other words, was never designed to answer the question clinicians actually need answered.

That question has also changed. The debate is no longer whether OSA belongs on the list of cardiovascular risk factors, which it clearly does, but which hypertensive patients stand to gain the most from being diagnosed and treated. Reframing the field around cardiovascular phenotype rather than respiratory severity is, in our view, a promising direction for sleep cardiovascular medicine, although it remains at present a hypothesis to be tested rather than a demonstrated advance [1,28].

Metrics such as hypoxic burden, the oxygen desaturation index, and ambulatory blood pressure phenotyping provide a richer picture of biological injury than the AHI alone, although only ambulatory blood pressure monitoring is currently supported by standardized thresholds and guideline endorsement. Paired with structured assessment of cardiac remodeling, vascular dysfunction, renal impairment, autonomic dysregulation, and the metabolic and hepatic abnormalities that frequently accompany OSA, this multidimensional view replaces a purely respiratory diagnosis with a map of cardiovascular risk [5,24,54].

That risk, moreover, is not one thing. Resistant hypertension, nocturnal hypertension, non-dipping and reverse-dipping blood pressure, masked hypertension, exaggerated morning surge, and advanced target-organ damage each carry distinct mechanisms, distinct prognoses, and distinct responses to treatment [20,34,39]. Treating them as a single “OSA-hypertension” entity obscures exactly the differences that should be driving clinical decisions.

The phenotype-oriented framework proposed in this review, built around screening, respiratory and cardiovascular phenotyping across five domains, risk categorization, and dynamic reassessment, is an attempt to give this shift a conceptual structure rather than simply argue for it. It is not meant to replace the AHI or existing hypertension guidelines, but to sit alongside them, giving clinicians a structured way to ask not only “how severe is this patient’s sleep apnea” but “what cardiovascular and metabolic phenotype does this patient have” [28,50].

Used this way, phenotyping has the potential to change practice, by identifying high-risk patients whose respiratory disease looks deceptively moderate, prompting earlier ambulatory blood pressure monitoring and target-organ assessment, sharpening the selection of patients most likely to benefit from CPAP, intensive antihypertensive therapy, or aggressive metabolic intervention, and giving multidisciplinary teams a common language. It also points toward where the field is heading, namely imaging, circulating biomarkers, wearables, and the kind of individualized treatment-effect modeling that machine learning has only just begun to make possible [64,65].

None of this is finished work. As stated at the outset of Section 6, the framework proposed here is a conceptual, author-derived synthesis rather than a validated risk-stratification instrument: it was not developed from patient-level data, and its three categories have not been calibrated or tested against clinical outcomes in an independent cohort. What is still missing is therefore prospective, multicenter validation: whether phenotype-guided management actually improves hard cardiovascular outcomes, not merely risk classification; standardized, reproducible measurement of hypoxic burden and related metrics; and trials designed around cardiovascular phenotype rather than respiratory severity as the entry criterion. These are substantial undertakings, but they are the logical next step, not a detour. Furthermore, several other limitations should be acknowledged. The available evidence is heterogeneous in study design, populations, diagnostic methods, and cardiovascular outcomes, and much of it derives from observational studies or post hoc analyses. Emerging metrics such as hypoxic burden, ODI, and T90 are promising but remain insufficiently standardized, with no universally accepted thresholds for routine clinical use.

The proposed cardiovascular phenotypes may overlap and change over time with aging, weight variation, treatment, adherence, and progression of cardiometabolic disease, which further limits the usefulness of any fixed classification and argues against interpreting the three categories as stable risk strata. In addition, the cardiovascular benefit of CPAP remains variable, and prospective trials specifically testing phenotype-guided management are still lacking. Until such trials are available, the framework should be regarded as a structured aid to clinical reasoning rather than as a basis for quantitative risk estimation or for treatment decisions that depart from current guideline recommendations.

Implementation is also limited by access to ABPM, advanced imaging, autonomic testing, biomarkers, and continuous monitoring, particularly in LMICs. Finally, many major OSA cohorts and intervention trials underrepresent women, younger individuals, ethnically diverse populations, and patients from LMICs, limiting the global generalizability of current phenotype-based models.

In conclusion, the assessment of patients with hypertension and obstructive sleep apnea appears to be entering a period in which the AHI remains essential for diagnosis but is increasingly recognized as insufficient for prognosis. The evidence reviewed here supports the view that cardiovascular and metabolic phenotype captures dimensions of risk that respiratory event frequency does not. Whether phenotype-guided risk stratification and treatment improve clinical outcomes, however, has not been tested prospectively, and the framework proposed in this review should therefore be regarded as an evidence-informed conceptual approach requiring validation rather than as a recommendation for practice. Establishing whether the shift from counting respiratory events to characterizing the whole of a patient’s cardiovascular and metabolic phenotype delivers measurable benefit is, in our view, the central task now facing the field.

## Figures and Tables

**Figure 1:**
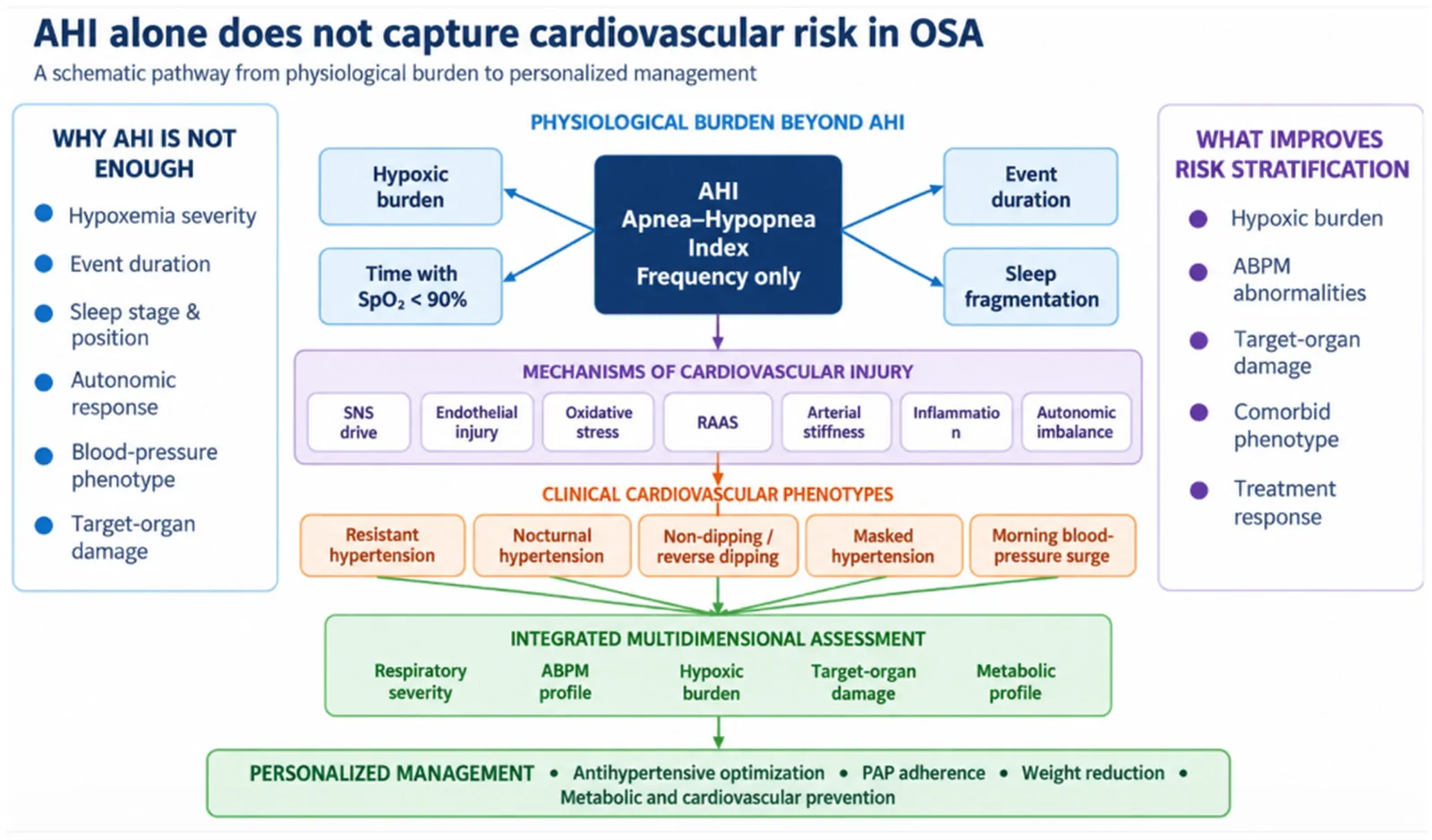
From respiratory disease to cardiovascular disease: conceptual evolution of obstructive sleep apnea. Conceptual overview of cardiovascular risk assessment in obstructive sleep apnea (OSA), from the limitations of the apnea-hypopnea index (AHI) to personalized management. The AHI captures respiratory event frequency but not hypoxemia depth, event duration, or blood pressure phenotype. Physiological burden metrics (hypoxic burden, ODI, T90, sleep fragmentation, arousal burden) feed into cardiovascular injury mechanisms (sympathetic activation, oxidative stress, RAAS activation, endothelial dysfunction, vascular inflammation, autonomic imbalance, metabolic dysfunction), which manifest as distinct clinical cardiovascular phenotypes and ultimately guide integrated, multidimensional risk assessment and personalized management.

**Figure 2:**
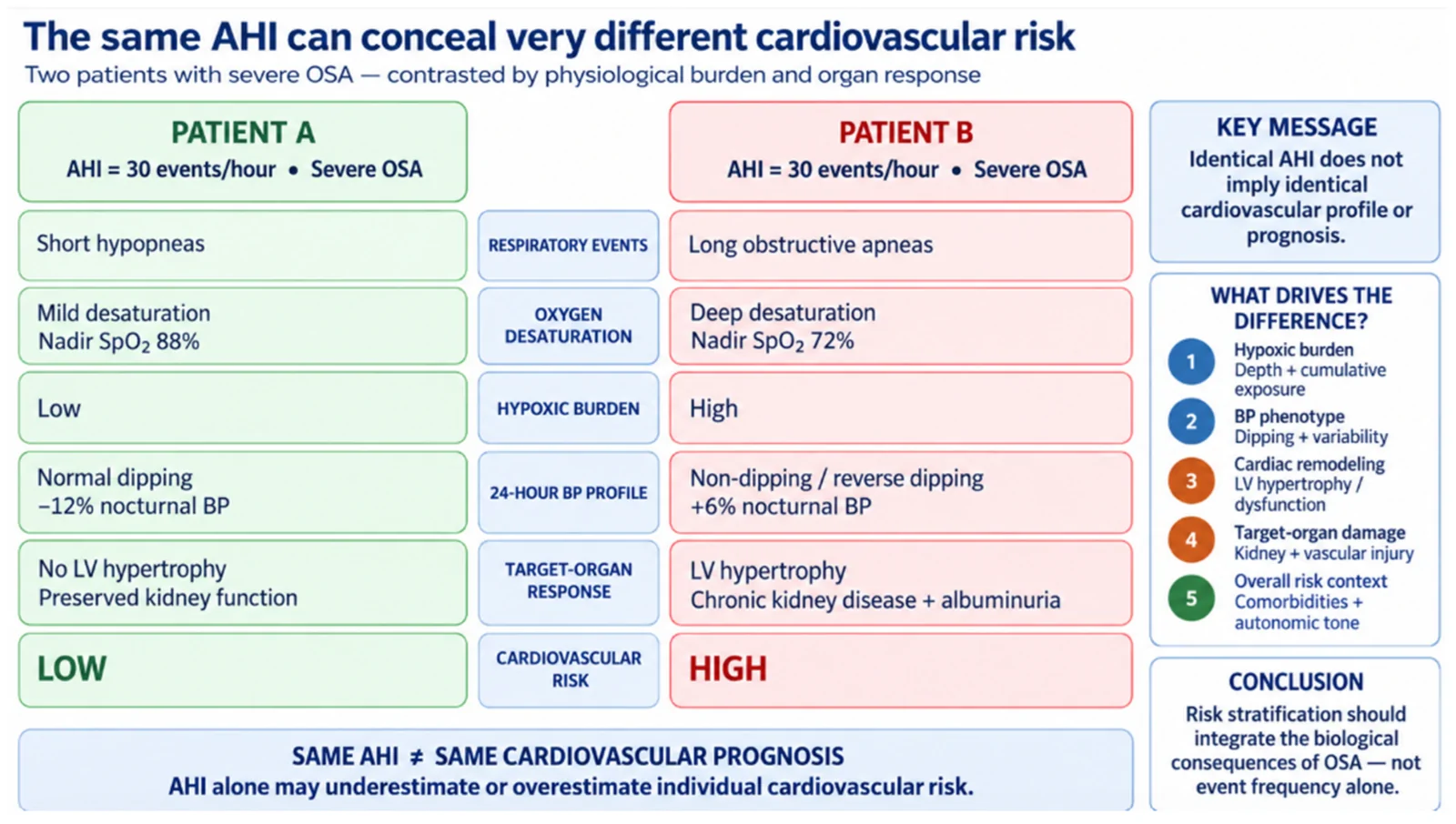
Illustrative comparison of two hypothetical patients with an identical apnea–hypopnea index (AHI = 30 events/h, severe OSA) but markedly different cardiovascular phenotypes. Patient A, with predominantly short hypopneas and mild desaturation, has low hypoxic burden, preserved nocturnal blood pressure dipping, no left ventricular hypertrophy, normal renal function, and low cardiovascular risk. Patient B, with predominantly long obstructive apneas and deep desaturation, has high hypoxic burden, a non-dipping/reverse-dipping profile, established left ventricular hypertrophy, chronic kidney disease, and high cardiovascular risk. The two patients are hypothetical and the values are illustrative throughout: they are not drawn from a reported case, from a patient cohort, or from any dataset analyzed by the authors, and were constructed to demonstrate that the AHI alone cannot distinguish between these two prognostically distinct presentations. No statistical inference should be drawn from the values shown. Green and red shading denote the low-risk and high-risk phenotypes, respectively; blue panels contain the compared parameters and the interpretive summary.

**Figure 3:**
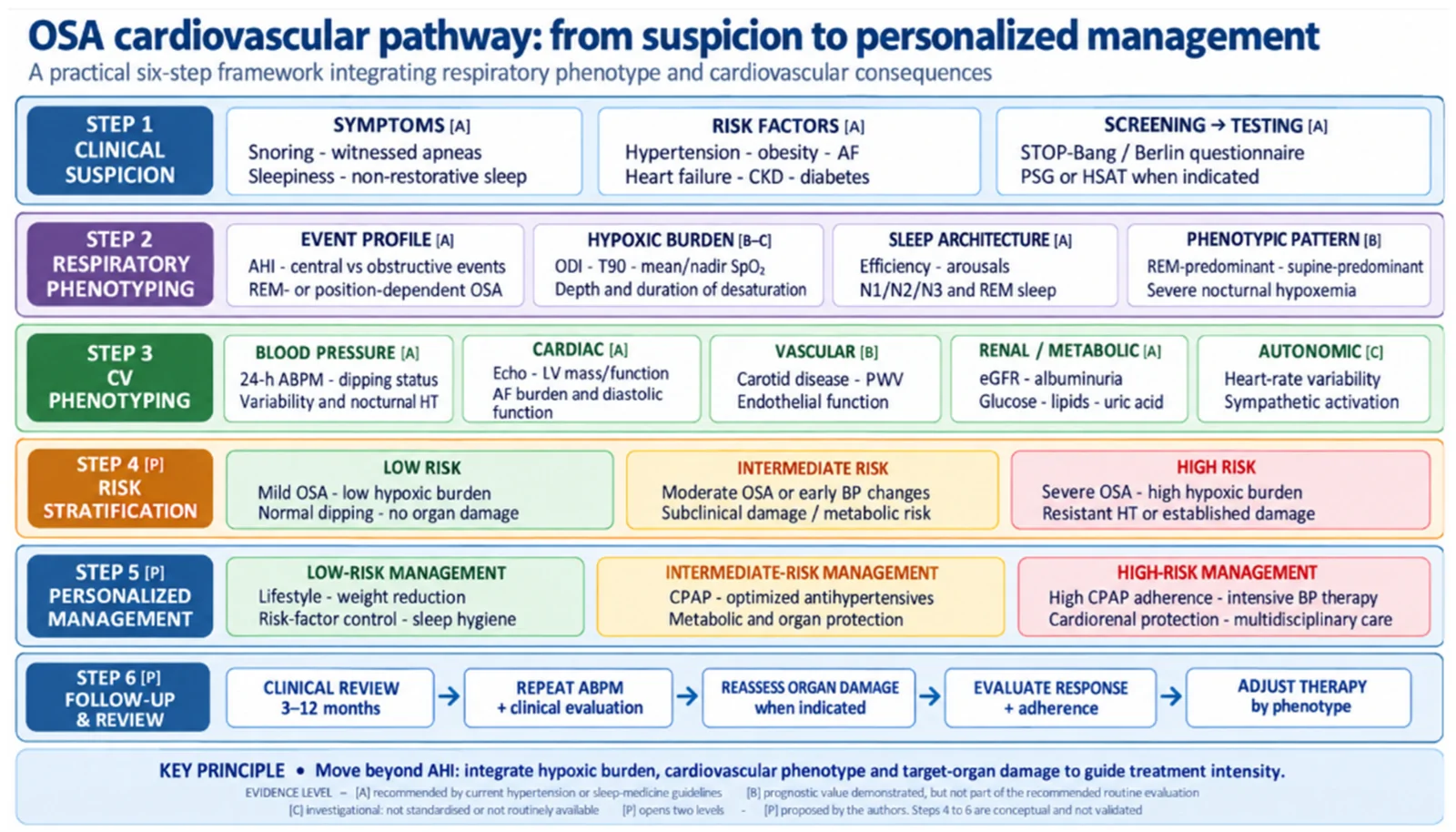
Proposed six-step clinical algorithm for cardiovascular phenotyping in hypertensive patients with obstructive sleep apnea, spanning initial clinical suspicion and screening (Step 1), respiratory phenotyping via polysomnography or home sleep apnea testing (Step 2), multidimensional cardiovascular assessment across blood pressure, cardiac, vascular, renal, metabolic, and autonomic domains (Step 3), risk stratification (Step 4), phenotype-guided personalized management (Step 5), and dynamic follow-up and reassessment (Step 6). Assessment components in Steps 1 to 3 carry an evidence-level marker: recommended by current hypertension or sleep-medicine guidelines; demonstrated prognostic value but not part of the recommended routine evaluation in this population; investigational, either not standardized or not routinely available; spanning two levels. Steps 4 to 6 carry the marker, indicating components proposed by the authors that are conceptual and have not been validated. These definitions are descriptive and are not derived from a formal evidence-grading instrument. In Steps 4 and 5, green, amber, and red shading denote low, intermediate, and high cardiovascular risk, respectively.

**Figure 4:**
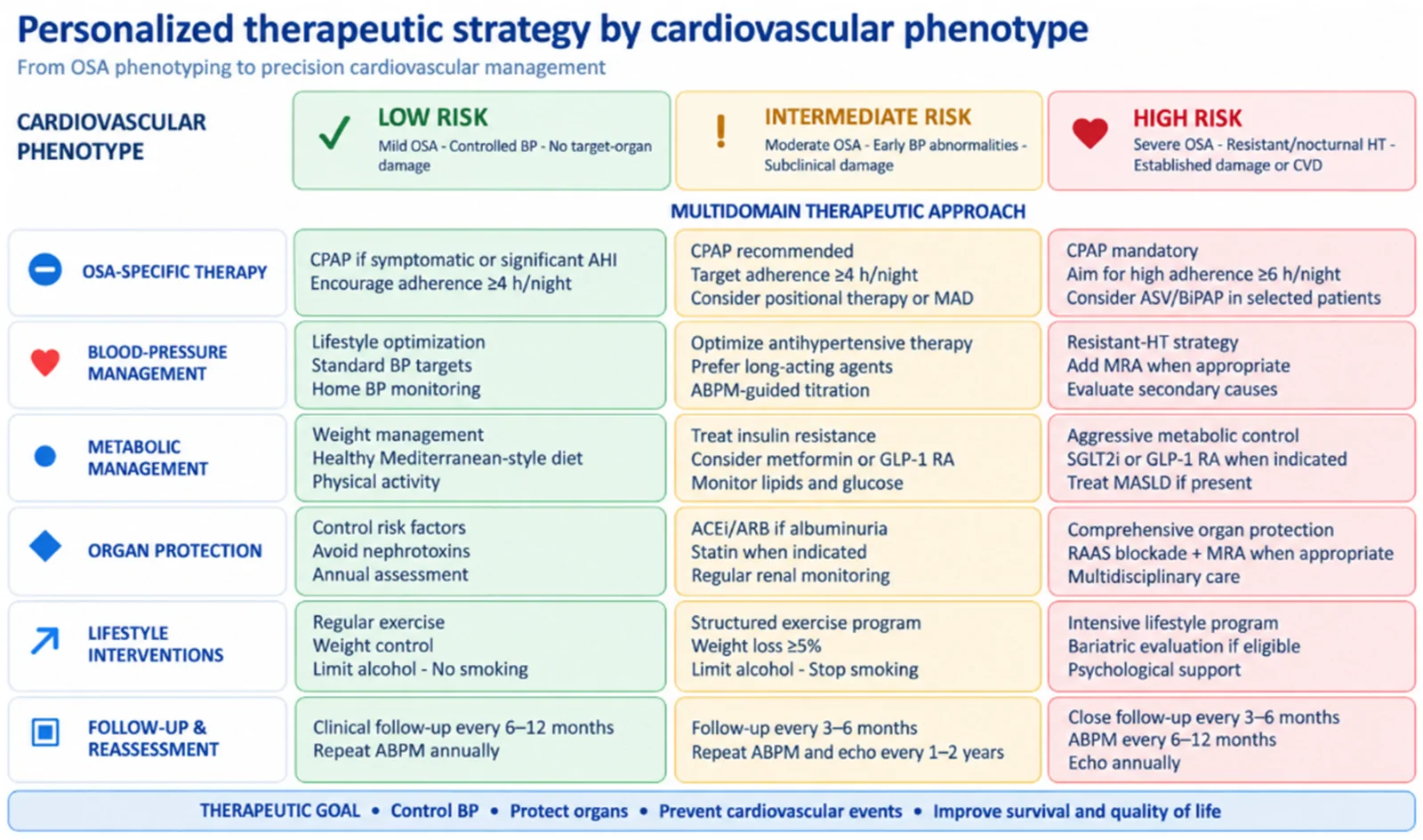
Personalized therapeutic strategy according to cardiovascular risk phenotype, spanning six management domains OSA-specific therapy, blood pressure management, metabolic management, organ protection, lifestyle interventions and follow-up, illustrating how therapeutic intensity and follow-up frequency escalate progressively from low to high cardiovascular risk.

**Table 1:** Comparative Performance of Conventional and Emerging Physiological Measures of Disease Severity in Obstructive Sleep Apnea.

Measure	Cardiovascular Prognostic Value	Strength of Evidence	Key Reference(s)	Main Knowledge Gap
AHI	Moderate	High (large prospective cohorts and meta-analyses)	[11]	Equal weighting of respiratory events regardless of hypoxic depth or duration
ODI	Moderate to High	High	[6,11]	Remains closely correlated with AHI; does not capture duration of desaturation
T90	High	Moderate to High	[6,11]	Captures duration but not depth of desaturation; oximeter and device variability affect reproducibility
Hypoxic burden	High	High (large multicohort studies)	[5,10,16]	Requires specialized software and is not yet implemented in routine clinical reporting; no consensus threshold for risk classification; prognostic superiority over the AHI is consistent for mortality and heart failure but has not been demonstrated for every cardiovascular endpoint
24-h ABPM	Very High	High	[19,25,26]	Underused in sleep clinics; rarely integrated into routine OSA evaluation

Ratings in this table are descriptive categories assigned by the authors on the basis of the evidence synthesized in Section 3. They do not represent the output of a formal evidence-grading instrument such as GRADE. Cardiovascular prognostic value was classified as Moderate when associations with cardiovascular outcomes were inconsistent across studies or attenuated substantially after adjustment; as Moderate to High when associations were consistent but added little incremental information beyond established indices; as High when independent associations with hypertension-mediated target-organ damage or intermediate cardiovascular endpoints were reproduced in several cohorts; and as Very High when independent associations with hard outcomes, such as cardiovascular mortality or incident heart failure, were demonstrated in more than one independent cohort, together with incremental predictive value over the AHI that was consistent across the cardiovascular endpoints examined. Strength of evidence was classified as Moderate when the supporting data derived mainly from single-center or cross-sectional studies; as Moderate to High when several prospective cohorts were available without pooled or multicohort confirmation; and as High when the finding was supported by large prospective cohorts, multicohort or meta-analytic data, or current guideline recommendations. In keeping with the narrative design of this review, no formal risk-of-bias appraisal was undertaken, and these categories should be read as an aid to comparison rather than as a graded recommendation.

**Table 2:** Cardiovascular Phenotypes of Hypertension in Obstructive Sleep Apnea, Grouped by Conceptual Domain.

Conceptual Domain	Phenotype	Defining Feature	Principal Mechanism	Distinctive Prognostic Marker	Key Reference(s)
Treatment-response category	Resistant hypertension	BP above target despite ≥3 antihypertensive classes including a diuretic, or controlled BP requiring ≥4	Sustained chemoreflex-mediated sympathetic and RAAS/aldosterone activation; aldosterone–airway-edema feedback loop	OSA prevalence > 70% among resistant hypertension clinic referrals; greatest BP benefit from CPAP with good adherence	[2,25,34,36,37]
Temporal ABPM pattern	Nocturnal hypertension	Elevated nighttime BP with normal office readings	Repetitive per-event sympathetic surges and intrathoracic pressure swings accumulating across the night	Correlates more closely with LVH, microalbuminuria, and carotid atherosclerosis than office BP; frequently missed without ABPM	[1,19,25,37]
Temporal ABPM pattern	Non-dipping/reverse-dipping	Loss of, or reversal of, the physiological 10–20% nocturnal BP decline	Persistent sympathetic tone, impaired baroreflex sensitivity, intermittent-hypoxia-driven oxidative stress and reduced NO bioavailability	Reverse dippers show greater left ventricular mass, arterial stiffness, and carotid IMT than normal dippers, independent of office BP	[22,39,40]
Measurement-discordance category	Masked hypertension	Normal office BP, elevated ambulatory or home BP	Nocturnal sympathetic activation persisting into daytime autonomic function	Cardiovascular mortality risk (HR ≈ 2.05) statistically comparable to sustained hypertension (HR ≈ 2.42)	[2,25,41]
Temporal ABPM pattern	Morning BP surge	Exaggerated rise in BP at the sleep–wake transition	Incomplete autonomic recovery from nocturnal chemoreflex/sympathetic activation	Associated with stroke, MI, sudden cardiac death, and plaque rupture; hypoxic burden tracks catecholamine surge more closely than AHI	[42,43,44]
Consequence-defined category	Hypertension-mediated target-organ damage	LVH, diastolic dysfunction, left atrial enlargement, arterial/renal/retinal/cognitive injury	Chronic intermittent hypoxia, oxidative stress, inflammation, neurohumoral activation	Present even at moderate AHI; independently defines a high-risk phenotype regardless of respiratory severity	[17,20,44,45,51]

The conceptual domain column indicates the level at which each entity is defined, as set out at the beginning of Section 4: temporal blood pressure patterns identified on ambulatory monitoring, categories defined by discordance between office and out-of-office measurement or by response to treatment, and a category defined by the structural consequences of sustained pressure and hypoxic injury. The six entities are not mutually exclusive; most high-risk patients present with a combination of two or more, as reflected in the risk stratification model proposed in Section 6 (Step 4). ABPM, ambulatory blood pressure monitoring; AHI, apnea-hypopnea index; BP, blood pressure; CPAP, continuous positive airway pressure; HR, hazard ratio; IMT, intima-media thickness; LVH, left ventricular hypertrophy; MI, myocardial infarction; NO, nitric oxide; OSA, obstructive sleep apnea; RAAS, renin-angiotensin-aldosterone system.

**Table 3:** Evidence Base, Prevalence, Metric Relationships and Clinical Actionability of the Six Cardiovascular Phenotypes.

Phenotype	Status	Reported Prevalence in OSA	Relationship with AHI and Hypoxic Burden	Does Identification Change Management?	Key Reference(s)
Resistant hypertension	Established; defined in current hypertension guidelines	The evidence base is asymmetric: OSA is reported in over 70% of patients referred for resistant hypertension, whereas the prevalence of resistant hypertension within unselected OSA cohorts is less well characterized. Meta-analysis confirms the association	Associated with AHI severity in a graded manner; comparative data for hypoxic burden are limited	Yes. Supports systematic screening for OSA in this population, and CPAP with good adherence produces measurable 24-h blood pressure reduction	[2,34,37]
Nocturnal hypertension	Established; defined by ambulatory criteria in current guidelines	Threshold-dependent and measurement-dependent. In a meta-analysis of ABPM studies in OSA, hypertension was detected in 66% of patients by ambulatory monitoring compared with 44% by office measurement; the ABPM figure fell to 59% when the study using nighttime criteria was excluded	Tracks hypoxemia metrics more closely than event frequency; effectively invisible to the AHI	Yes. Detection requires ABPM and alters both the diagnosis of hypertension and the intensity of treatment	[17,28,34,52]
Non-dipping and reverse-dipping	Established ambulatory pattern with an extensive literature independent of sleep medicine	Pooled prevalence 59.1% (95% CI 52.0–65.0) across 14 studies and 1562 patients with OSA, with between-study values ranging from 36% to 90%	More frequent with increasing OSA severity; the association strengthens when patients with mild OSA are excluded	Partly. Supports targeting nocturnal blood pressure, although the benefit of chronotherapy remains unsettled in recent randomized trials	[53]
Masked hypertension	Established; defined in current hypertension guidelines	Cumulative prevalence of approximately 34% in a meta-analysis of ambulatory monitoring studies in OSA	Not well characterized against either metric; detection depends on ambulatory monitoring rather than on respiratory indices	Yes. Reclassifies an apparently normotensive patient as hypertensive and triggers antihypertensive treatment	[52]
Morning blood pressure surge	Established ambulatory parameter, although competing definitions limit comparability across studies	Not established in OSA populations	In the one direct comparison available, hypoxic burden tracked the catecholamine surge more closely while the AHI remained the stronger predictor of morning diastolic blood pressure (Section 4.5)	Limited. No trial has demonstrated that targeting the morning surge improves outcomes in this population	[42]
Hypertension-mediated target-organ damage	Established; recognized as a risk modifier in current hypertension guidelines	Varies substantially with the imaging modality and the diagnostic criteria applied	Correlates more closely with cumulative hypoxic burden than with event counts in cross-sectional analyses; present even at moderate AHI	Yes. Its presence reclassifies cardiovascular risk independently of measured blood pressure and raises treatment intensity	[17,20,44]

None of the six entities is novel in itself; each is an established clinical category with a literature independent of sleep medicine, as set out at the beginning of Section 4. Prevalence estimates are drawn from populations of differing composition and are not directly comparable across rows. ABPM, ambulatory blood pressure monitoring; AHI, apnea-hypopnea index; CI, confidence interval; CPAP, continuous positive airway pressure; OSA, obstructive sleep apnea.

**Table 4:** Proposed Conceptual Grouping of Cardiovascular Risk Categories and Corresponding Management Intensity.

Risk Phenotype	Key Defining Features	Primary Therapeutic Focus	Follow-Up Intensity	Key Reference(s)
Low	Controlled hypertension, preserved nocturnal dipping, limited hypoxemia, no target-organ damage, favorable metabolic/hepatic profile	Lifestyle optimization; CPAP mainly for symptomatic benefit	Routine, periodic reassessment	[2,17,24]
Intermediate	Moderate hypoxemia, non-dipping profile, early LV remodeling, obesity, insulin resistance or early hepatic steatosis, mild CKD	Guideline-based antihypertensive optimization (RAAS blockers, MRA when indicated); CPAP adherence; disease-modifying weight loss; SGLT2i/GLP-1RA when metabolically indicated	Closer surveillance; this window is where progression is still preventable	[1,22,24,54]
High	Resistant/severe nocturnal hypertension, reverse dipping, marked hypoxic burden, advanced LVH, AFib, CKD, heart failure, diabetes, obesity	ABPM-guided intensive BP control; CPAP optimization; aggressive cardiometabolic therapy; arrhythmia- and heart-failure–specific management	Multidisciplinary, individualized, most frequent	[50,59,63,66,71]

The categories in this table are author-defined and illustrative; they have not been validated against clinical outcomes. The interventions listed under “Primary Therapeutic Focus” are drawn from current hypertension, sleep-medicine, diabetes and cardiovascular guidelines. Their allocation across the three categories, and the graduation of follow-up intensity shown in the final column, are proposed by the authors and have not been prospectively validated.

## Data Availability

No new data were created or analyzed in this study. Data sharing is not applicable to this article.
